# Cognitive components of a mathematical processing network in 9-year-old children

**DOI:** 10.1111/desc.12144

**Published:** 2014-02-23

**Authors:** Dénes Szűcs, Amy Devine, Fruzsina Soltesz, Alison Nobes, Florence Gabriel

**Affiliations:** 1 Department of Psychology, Centre for Neuroscience in Education, University of CambridgeUK; 2 Department of Psychiatry, University of CambridgeUK

## Abstract

We determined how various cognitive abilities, including several measures of a proposed domain-specific number sense, relate to mathematical competence in nearly 100 9-year-old children with normal reading skill. Results are consistent with an extended number processing network and suggest that important processing nodes of this network are phonological processing, verbal knowledge, visuo-spatial short-term and working memory, spatial ability and general executive functioning. The model was highly specific to predicting arithmetic performance. There were no strong relations between mathematical achievement and verbal short-term and working memory, sustained attention, response inhibition, finger knowledge and symbolic number comparison performance. Non-verbal intelligence measures were also non-significant predictors when added to our model. Number sense variables were non-significant predictors in the model and they were also non-significant predictors when entered into regression analysis with only a single visuo-spatial WM measure. Number sense variables were predicted by sustained attention. Results support a network theory of mathematical competence in primary school children and falsify the importance of a proposed modular ‘number sense’. We suggest an ‘executive memory function centric’ model of mathematical processing. Mapping a complex processing network requires that studies consider the complex predictor space of mathematics rather than just focusing on a single or a few explanatory factors.

## Introduction

Mathematics likely builds on several cognitive abilities (Passolunghi, Vercelloni & Schadee, 2007; Passolunghi & Lanfranchi, 2012; Krajewski & Schneider, 2008; Geary, 2012; Swanson & Jerman, 2006) implemented by an extended neural network of the brain (Supekar, Musen & Menon, 2009; Bressler & Menon, 2010; Menon, 2010; Fias, Menon & Szűcs, 2013; Goswami & Szűcs, 2011; Dowker, 2005). However, to date there have been only a few studies considering several of these abilities in a single framework. Further, in recent years the idea of a potentially biologically based ‘number sense’, a non-symbolic magnitude representation has received a lot of attention as an explanatory factor behind mathematical performance (Dehaene, 1997). Some argue that mathematical skill is more related to a non-symbolic magnitude representation while others claim that the key is a link between the magnitude representation and symbols (see De Smedt & Gilmore, 2011, and Noël & Rouselle, 2011, for overviews). Hence, it is important to compare the predictive power of number sense variables to those of more general variables with robust methods in a single framework. Here we report such a study which relied on distribution independent permutation testing and confidence interval estimation of correlation and regression models contrasting the predictive power of several variables on mathematical performance in 9-year-old children.

A large number of studies strongly associated short-term memory (STM) and working memory (WM) with mathematical achievement by a large number of studies (see reviews in Raghubar, Barnes & Hecht, 2010; Gathercole, Pickering, Night & Stegmann, 2004; Swanson & Jerman, 2006; Geary, 2012; Passolunghi, Mammarella & Altoè, 2008; Passolunghi & Mammarella, 2010; Passolunghi & Siegel, 2001; Simmons, Willis & Adams, 2012). STM only requires the maintenance of information while WM also requires an additional processing component besides maintenance. Most studies have relied on Baddeley's WM model (1986) and have assumed that the additional processing component relies on domain-general central executive (CE) function. Hence, most studies have tested WM with verbal tasks only. However, evidence is now accumulating that verbal and visual WM function can dissociate (Shah & Miyake, 1996; Jarvis & Gathercole, 2003; Klauer & Zhao, 2004) and in fact may differently relate to mathematical competence as several studies testing both verbal and visual memory found that only visual but not verbal WM performance discriminates children with poor and typical mathematical achievement (White, Moffitt & Silva, 1992; Andersson & Östergren, 2012; Szűcs, Devine, Soltesz, Nobes & Gabriel, 2013a; Kyttälä & Lehto, 2008). One study tested children with poor mathematical or poor reading achievement as well as children with a combined deficit and found that visual STM was related to poor mathematical achievement, and poor reading achievement was linked to poor verbal STM while the double deficit group had poor verbal WM (Schuchardt, Maehler & Hasselhorn, 2008; see also Van der Sluis, van der Leij & de Jong, 2005). Recent studies have further confirmed the specific importance of visual STM and WM for mathematical development (Passolunghi & Lanfranchi, 2012; Szűcs *et al*., 2013a). On the other hand, results are compatible with the wider literature, e.g. studies with dyslexic children and poor readers have reported impaired verbal STM (Helland & Asbjornsen, 2004; Hecht, Torgesen, Wagner & Rashotte, 2001; Schuchardt *et al*., 2008; Pimperton & Nation, 2010) and children with specific language impairment have very poor verbal STM and show weak counting and calculation skills (Donlan, Cowan, Newton & Lloyd, 2007). Hence, poor verbal memory primarily seems to be connected to poor reading while poor visual memory seems more directly related to poor mathematical function. The above results suggest that it is important to test verbal and visual aspects of both STM and WM function.

Phonological skill is another important domain to examine. Reading and math ability are known to be associated with each other (Jordan, Wylie & Mulhern, 2010). For example, recently in a sample 1004 British 7- to 10-year-old children, Devine, Soltesz, Nobes, Goswami and Szűcs (2013) reported *r* = 0.626 correlation between standardized reading and mathematics scores. Phonological ability is known to be important for reading skill (Goswami, 2011; Ziegler & Goswami, 2005; Bradley & Bryant, 1983) and it can also be expected to play a role in arithmetic as written computational problems are typically coded into speech-based representations during solution and often problems are communicated in speech. Furthermore, verbally coded solutions to multiplication and addition problems are likely to be retrieved from long-term phonological memory rather than computed on demand (Ashcraft, 1995; Ashcraft & Stazyk, 1981; Ashcraft & Bataglia, 1978) and counting strategies strongly build on learnt verbal associations. In fact, Leather and Henry (1994) reported strong correlations between phonological awareness measures and arithmetic test scores. Hecht *et al*. (2001) found that phonological memory (verbal STM/WM), the rate of access to phonological codes in long-term memory (naming digits and letters) and phonological awareness were strongly associated with computational ability and overall phonological skill nearly completely explained the relationship between reading and computational ability. More recently, Swanson and Beebe-Frankenberger (2004) and Simmons, Singleton and Horne (2008) reported similar results. Importantly, however, studies have also reported negative results. de Jong and van der Leij (1999), Passolunghi *et al*. (2007) and Passolunghi *et al*. (2008) found no relation between phonological ability and math achievement. Hence, overall it is not clear how and when during development phonological ability contributes to mathematical development.

Non-verbal intelligence also seems strongly related to mathematical achievement. For example, in a longitudinal study from kindergarten to grade 2, de Jong and van der Leij (1999) found that non-verbal intelligence, word knowledge, and phonological awareness were important predictors of initial mathematical skill. Similarly, Kyttala and Lehto (2008) found that non-verbal fluid intelligence was the best predictor of *mental arithmetic*, explaining 23% of the variance, while Raven's Progressive Matrices explained 37% of the variance in geometry problems. Non-verbal intelligence may partially depend on spatial skills which have been reported to be weak in children with poor mathematical achievement (Rourke & Conway, 1997; Rourke, 1993). Spatial processes can be potentially important in mathematics where explicit or implicit visualization is required, like when imagining operations along the number line or visualizing functional relationships. In fact, Rourke (1988) suggested that specific poor mathematical achievement can be related to so-called non-verbal learning difficulties characterized by poor visuo-spatial organization skill. Of course, as the above studies demonstrate several other factors relate to mathematical achievement besides spatial skills and poor spatial skills may also relate to weak visuo-spatial STM/WM.

Several studies have found that mathematical skill correlates with general executive functioning and task switching (e.g. Van der Ven, Kroesbergen, Boom & Leseman, 2012; Bull, Espy & Wiebe, 2008; Conlin, Gathercole & Adams, 2005). Several studies have used the trail-making A and B tasks as measures of executive functioning and task switching and have shown that performance on these tasks is correlated with mathematical skill (Soltesz, Szücs, Dékány, Márkus & Csépe, 2007; McLean & Hitch, 1999; White *et al*., 1992; Szűcs *et al*., 2013a). Others reported that mathematical achievement was related to attentional function (Swanson, 2011; Ashkenazi, Rosenberg-Lee, Tenison & Menon, 2012; Hannula, Lepola & Lehtinen, 2010) and several studies found that inhibitory function was related to the level of mathematical development (Bull & Scerif, 2001; Bull, Johnston & Roy, 1999; Passolunghi, Cornoldi & De Liberto, 1999; Passolunghi & Siegel, 2004; McKenzie, Bull & Gray, 2003; Espy, McDiarmid, Cwik, Stalets, Hamby & Senn, 2004; Blair & Razza, 2007; Swanson, 2011). Task-switching (control), attentional and inhibition processes seem very important for mathematics because they coordinate which items of interest receive processing and when and in what order they enter processing. Such functions are probably very important in calculations which require the continuous selection and coordination of several processing steps and items in memory. In fact, task-switching, inhibitory, attentional and working memory processes may all be intricately intertwined and form the core of ‘central executive’ memory processes (Hasher & Zacks, 1988; Miyake, Friedman, Emerson, Witzki, Howerter & Wager, 2000).

Recently, some have suggested that mathematical achievement strongly relates to a so-called number sense, a proposed domain-specific intuition for magnitude (Dehaene, 1997). Number sense has typically been measured using non-symbolic magnitude discrimination tasks where children decide which of two dot patterns is more numerous. In such a task Mazzocco, Feigenson and Halberda (2011) set up several regression models and found that magnitude discrimination ability predicts performance on standardized mathematics tests even when spatial memory performance is considered. However, beta values were not communicated and, more critically, mathematics and magnitude discrimination performance were measured in grades 8 and 9 while memory performance scores were determined in grade 3. Considering the 6-year gap between taking measures, the validity of the analyses is not clear. In another study, Piazza, Facoetti, Trussardi, Berteletti, Conte, Lucangeli, Dehaene and Zorzi (2010) found that non-symbolic comparison performance moderately correlated with two measures of symbolic number comparison (*R*^2^ = 0.17; *p* = .049) and it was noted that this relationship remained even after controlling for verbal IQ. However, other math performance measures were not correlated with non-symbolic discrimination and other variables were not controlled. Halberda, Mazzocco and Feigenson (2008) also reported ‘retrospective prediction’ of math performance from performance in a non-symbolic decision task. However, in this study non-symbolic comparison was actually measured well after math performance. That is, better math performance in this study may have been a cause of better non-symbolic comparison performance rather than vice versa (see full argument and summary of studies in Noël & Rouselle, 2011 and Soltész, Szűcs & Szűcs, 2010). Besides, the above studies typically use hierarchical regression analysis which relies on (very) strong model assumptions that can greatly bias analysis outcomes. Overall, there is a clear need for a study that considers the predictive power of number sense variables on mathematical achievement in the context of several other variables.

In this study we contrasted the predictive power of several cognitive abilities, including various measures of the number sense, on mathematical skill in nearly 100 children. We tested both verbal and visual STM and WM, had a measure of phonological decoding, two measures of non-verbal intelligence (Raven's CPM and WISC Block Design), tests of executive functioning and task switching (Trail-making A and B). We had measures of spatial orientation ability, knowledge of spatial symmetry, mental rotation, finger knowledge, a measure of sustained attention and response inhibition (stop signal task), and measured baseline simple RT in a target detection task. We measured spatial bias using a line bisection task and determined several parameters (accuracy, RT and coefficient of variation) of three proposed measures of the number sense (non-symbolic and symbolic number comparison and subitizing). We also measured dot enumeration performance in the counting range (4–6 dots). We used two standardized tests of mathematical operations as outcome measures and a standardized reading test as a control outcome measure. Hence, we tested whether our preferred model specifically predicts mathematics achievement. We used robust permutation based bootstrap correlation and regression analyses which are not subject to any distributional assumptions. Bootstrap confidence interval estimation is also useful because it can quantify uncertainty in parameter estimates (correlation coefficients and beta values) which is particularly important in simultaneous regression analyses where several variables ‘compete’ to explain a share of variance. Hence, confidence intervals give a good indication of the robustness/stability of particular parameters which is especially important in psychological data where several variables may be interrelated.

## Methods

For complete description see the Supplementary Methods.

### Participants

Here we report data from 98 children recruited from Year 3 and Year 4 classes of schools in Cambridgeshire, Hertfordshire and Essex in the United Kingdom. There were 51 girls (mean age = 8.9 years; *SD* = 0.5; range = 7.8 to 10.3 years) and 47 boys (mean age = 9.0 years; *SD* = 0.5; range = 8.3 to 10.5 years). The socioeconomic status score was 3.6±1.9 in girls (mean±*SD*) and 3.8±2.0 in boys (see detailed scoring in Supplementary Methods). Children with at least normal reading skill (standard reading score > 85) were invited to take part in the study from a sample of 1004 British children described in detail in Devine *et al*. (2013). The parents of 104 children gave consent to taking part in the detailed study reported here. Children completed approximately 7 hours of testing across several testing sessions, and 95 to 98 children had available scores along all measures examined in crucial regression models reported in the current paper (98 for the best models and 95 for number sense variable focused models). Data for these children are reported here.

### Measures and procedure

#### Standardized tests

Children were initially tested using standardized group mathematics and reading tests which were administered to whole classes. The math test used was the Mathematics Assessment for Learning and Teaching test (MaLT; Williams, 2005). Reading ability was assessed using the Hodder Group Reading Test II, levels 1 and 2 (HGRT-II; Vincent & Crumpler, 2007).

Children were individually administered an additional standardized measure of mathematical ability (the Numerical Operations subtest of Wechsler Individual Achievement Test (WIAT-II; Wechsler, 2005)). Math performance (hereafter: math) in this study is characterized by the mean score on the MaLT and the WIAT Numerical Operations subtest. There was a standardized measure of reading ability (WIAT-II Word Reading subtest) and a standardized measure of phonological decoding (WIAT-II Pseudoword Decoding subtest), and two IQ tests (the Raven's Coloured Progressive Matrices (Raven's CPM; Raven, 2008) and a short form of the Wechsler Intelligence Scale for Children – 3rd Edition (WISC-III; Wechsler, 1991)). The WISC-III short form included the Block Design (non-verbal) and Vocabulary (verbal) subtests. This combination of subtests has the highest validity and reliability of the two-subtest forms (*r*_tt_ = .91 *r* = .86; Table L-II, Sattler, 1992). Socioeconomic status was estimated from parents’ education levels and occupations. Children were also administered five subtests of the Automated Working Memory Assessment (AWMA; Alloway, 2007), which included two measures of verbal short term memory (STM): Digit Span and Word Recall; one measure of visuo-spatial STM: Dot Matrix; one measure of verbal working memory: Listening Span (recall and processing scores); and one measure of visuo-spatial working memory: Odd One Out (Odd-one-out; recall and processing scores).

#### Experimental tasks

##### Trail-making task

Trail-making tests A and B were administered. Each received a score (2 = no errors or self-corrected, 1 = one error, 0 = two or more errors), and solution speed was measured in seconds.

##### Finger knowledge

Children were asked to identify, with their eyes closed, which finger had been touched by the experimenter using the eraser end of a pencil. Children were familiarized with finger names prior to the task. The experimenter touched the children's fingers in a randomized order and responses were recorded using a voice recorder. Each response received a score (1 = correct, 0 = incorrect).

##### Line bisection

Children bisected 16 lines of varying length. Children were instructed to mark where they thought the ‘halfway’ point of each line was. Lines were presented individually on strips of paper. Lines varied with respect to the alignment with the midline of the strips of paper. The total time to bisect the 16 lines was recorded. The distance from the leftmost point of the line to the point where the children made their midpoint estimate was measured to the nearest mm using a ruler. The difference between the actual midpoint and the estimate was calculated and recorded for each item.

##### Mental rotation

Three separate worksheets with different stimuli types (objects/animals, letters and hands) were presented to the children; each worksheet had seven items. For each item within a worksheet, a target stimulus was presented, along with three comparison stimuli, two of which were mirror images (distractors) and one was identical to the target. All three comparison images were rotated by various angles. The children were required to identify and circle the stimulus identical to the target. Children's accuracy and time to complete all seven items was recorded for each worksheet.

##### Spatial symmetry

Children were presented with two pages which contained six half-drawn shapes against a grid background. A dashed line indicated the line of symmetry. Children were required to draw the other half of the shape for each item. Shapes (and lines of symmetry) were presented vertically on one page and horizontally on the other. The total time to complete the 12 shapes was recorded and the accuracy of items was scored with one point for every correct line segment.

##### Spatial orientation

Children were presented with a map containing different items such as a tree, car, cat and traffic light. Children were required to imagine themselves in this space, and to imagine they were standing next to one item, and facing another item. The children were then required to estimate the direction of a third item and draw the location of this item in relation to the other two items on a piece of paper. Responses were recorded as correct if they fell within ±20 degrees of the correct location. Correct responses received a score of 1, with a maximum of 6 points available. It is important to note that this scale was not standardized and we could not determine the reliability of this scale, which is a limitation of our study. Future research should use a proven reliable measure of this construct.

##### Simple RT

Children pressed a key in response to a white square which appeared after 1000, 2500 or 4000 ms. There were 60 trials.

##### Sustained attention

Children were required to attend to a stimuli stream (letters) and to detect a target sequence (A B C) and to withhold responses to other sequences containing the target letters (‘deceiver’ trials; e.g. A B D etc.) or sequences containing no target letters (‘non-target’ trials; e.g. D H F). The number of hits and misses for targets, the RT for target hits, the number of correct rejections and false alarms for deceivers and non-target trials were recorded. Children were presented with 80 triads of the three different trial types.

##### Stop signal task

A white arrow, pointing left or right, was shown on a black background in the middle of the screen. The arrow was followed by either a sound, the stop signal, or there was no sound. Children were required to indicate the direction of the arrow using a key press during ‘go’ trials, and to withhold their responses during ‘stop’ trials. The time delay until the stop sound was dynamically varied between 0 and 1000 ms depending on performance (see Supplementary Methods). The ratio of ‘go’ and ‘stop’ trials was 2:1. Children completed three blocks of 60 trials. For each trial we measured RT, Stop Signal RT (defined as the RT − average stop signal delay), and the number of times the child responded to the arrow incorrectly.

##### Number sense measure 1: Non-symbolic magnitude comparison

Two sets of black dots were presented simultaneously on a white background. The children's task was to decide which set contained more dots and press the button on the side of the larger set. Dot size was varied between sets. There were four blocks of 32 stimuli. See more details in Supplementary Methods.

##### Number sense measure 2: Symbolic magnitude comparison

Children decided whether visually presented digits were smaller or larger than 5. Children pressed a button on the keyboard with their left hand if the number was smaller than 5 and another button with their right hand if the number was larger than 5. Two blocks of 40 stimuli were presented.

##### Number sense measure 3: Subitizing

Arrays containing one to six black dots appeared on a white background and children were instructed to say the number of dots as quickly as possible. Dot stimuli were presented in canonical and, where possible, non-canonical arrangements. Two blocks of 30 trials were presented. RTs were measured using a voice-key.

### Statistics

To start with, zero-order and partial correlations were studied. Partial correlations controlled for the influence of three IQ variables: WISC Vocabulary, WISC Block Design and Raven's CPM (hereafter: Raven). Other variables were considered to have a robust relationship with math if their correlation remained even after controlling for the three IQ variables. Highly intercorrelated variables were averaged in order to avoid problems with multicollinearity (see Results).

In order to assess the robustness of correlations, a bootstrap procedure determined empirical 95% confidence intervals for correlations. Bootstrap and permutation procedures followed Chihara and Hesterberg (2011) and Fox (2008). These procedures do not rely on any assumptions regarding the distribution of variables. In all, 100,000 bootstrap samples were taken with replacement, the correlation coefficient was computed for each sample and confidence limits were determined using values at the 2.5% and 97.5% centiles. When computing bootstrap confidence intervals, correlations can be considered robust if their confidence interval does not include zero. That is, this procedure offers another criterion for establishing the significance of correlations besides traditionally used *p* values.

In order to determine the importance of individual predictors, simultaneous multiple linear regression was used throughout this study. The order of entry into models is irrelevant in simultaneous regressions (unlike in hierarchical regression). The procedure finds an optimally weighted sum of predictor variables for predicting the dependent variable. We were interested in the relative importance of variables, hence standardized β values will be reported rather than unstandardized Beta (B) coefficients.

The modelling attempt started with the potential predictor variables showing significant partial correlations with math. These variables were entered into the regression and variables with significant β values were identified. We tested whether non-significant predictor variables can become significant predictors when adding them to the model one by one. This was achieved by adding a single, previously non-significant variable to the model with significant predictors one by one. None of the non-significant predictors became significant.

After a best model was identified, further procedures tested whether adding IQ variables (WISC Vocabulary, WISC Block Design and Raven) to the regression model changed significant predictors and improved fit. In order to determine potential gender differences, another analysis also added Gender as a dummy variable to the best model. In order to get a measure of any potentially remaining multi-collinearity problems in regression models the variance inflation factor (VIF; see e.g. Cohen, 2003; O'Brien, 2007) was computed from the overall intercorrelation tables of all variables tried in various models. Typically, VIF values larger than 5 or 10 are considered to indicate multi-collinearity problems. All VIF values we measured were smaller than 2.02.

In order to study the robustness of models, bootstrap confidence intervals were computed for the β values of each predictor. To this end, 100,000 bootstrap samples were generated with replacement. The confidence intervals of robustly significant variables should not include zero. In addition, permutation testing of the significance of β values and whole models was also carried out. To this end, 100,000 permutations were generated without replacement. That is, the order of rows of the dependent variable was kept fixed and the order of the rows of the predictor variables was permuted 100,000 times and a regression was run for each 100,000 random samples. The significance level can then be determined by assessing the extremity of regression parameters relative to the 100,001 samples (100,000 random samples plus the original sample). Generating 100,000 random samples allows for determining *p* values with 10^-5^ precision. Analyses were done in Matlab 8.1 (2013a) (www.mathworks.com) and in R 2.14.1 (www.r-project.org).

## Results

### Zero-order and partial correlations

There was a strong relation between math and intelligence measures: WISC Block Design (*r* = 0.53; *p* < .001), Raven (*r* = 0.49; *p* < .001) and WISC Vocabulary (*r* = 0.52; *p* < .001). Hence, other variables were considered to have a robust relationship with Math if their correlation remained even after controlling for WISC Block Design, Raven and WISC Vocabulary scores. Significant partial and zero-order correlations are shown in Table1. For completeness, Table1 shows correlations for all tests including the non-significant partial correlations with the Hodder Group Reading Test and AWMA Word Recall (verbal STM). There were no significant partial or zero-order correlations with simple RT (*r* = −0.13), finger knowledge (*r* = 0.05), line bisection deviation score (*r* = 0.12) or RT (*r* = −0.016). There were no significant partial correlations with symmetry total score (*r* = 0.16) or RT (*r* = 0.06) and mental rotation score (*r* = −0.02) or RT (*r* = −0.11). However, zero-order correlations with the symmetry total score (*r* = 0.43, *p* < .001) and mental rotation accuracy score (*r* = 0.29, *p* < .01) were significant. Hence, these measures will also be investigated further later.

**Table 1:** Zero-order and partial correlations between math and standardized (when applicable) and raw test scores. Test abbreviations: HGRT: Hodder Group Reading Test. Phon.Dec: Phonological decoding. List. span recall/processing: Listening Span. OOO: Odd-one-out test. Spatial Orient: Spatial Orientation. Sust.Att: Sustained attention. Stop sig. Corr.R: Stop signal task Correct Rejection rate. Trail-A time: Trail-making A time. Sym.Com: symbolic number comparison accuracy score. Significance levels: red: p < .001; magenta: p < .01; blue: p < .05

*R*	Test score	HGRT	WIAT Word Reading	WIAT Phon.Dec.	Digit Recall	List. Span Recall	List. Span Proc.	Word Recall	Dot Matrix	OOO Proc.	OOO Recall	Spatial Orien.	Sust. Att.	Stop Sig. Corr.R.	Trail-A Time	Symbol. Com.
Partial	Standardized	0.14	0.43	0.31	0.32	0.26	0.22	0.14	0.48	0.40	0.36	–	–	–	–	–
	Raw	0.18	0.36	0.28	0.28	0.24	0.29	0.13	0.43	0.35	0.32	0.32	0.21	0.23	−0.22	0.28
Zero-order	Standardized	0.37	0.56	0.44	0.46	0.47	0.45	0.30	0.57	0.60	0.59	–	–	–	–	–
	Raw	0.45	0.50	0.40	0.42	0.44	0.47	0.28	0.53	0.55	0.56	0.45	0.36	0.16	−0.35	0.25

None of the number sense slope or Coefficient of Variation (COV) measures showed significant partial correlations in accuracy or in RT (0 ≤ *r* ≤ 0.22; non-symbolic comparison, symbolic comparison, subitizing and counting range dot enumeration). There were no correlations with the percent of correct solutions in the dot enumeration and non-symbolic magnitude discrimination task (0 ≤ *r* ≤ 0.15). The sole number sense related measure which showed significant partial correlation with math was the percentage of correct solutions in the symbolic number discrimination task (see Table1). Some number sense measures did show significant zero-order correlations with math. These will also be investigated further below.

### Averaging highly correlated variables

Odd-one-out recall and Odd-one-out processing scores were highly correlated (*r* = 0.96). Hence they were averaged to form a visual WM score. Listening Span Recall and Listening Span Processing scores were highly correlated (*r* = 0.95). Hence, they were averaged to form a Verbal WM score. The WIAT Word Reading and WIAT Pseudo-word reading scores were highly correlated (*r* = 0.77). Hence, they were averaged to form a Phonological Decoding score. Full zero-order and partial correlation tables for all relevant measures are shown in Supplementary Table2. While there were no significant partial correlations between math and most number sense measures except symbolic number comparison RT, it is theoretically important to see their relation with math and related variables. Hence, correlations are also shown for all number sense variables, except for slope measures which did not show any zero-order correlation with math (all: *r* ≤ 0.12).

**Table 2:** Descriptive statistics. Phon.Dec.: Phonological decoding (WIAT). Spatial Or.: Spatial Orientation. Trail-A time: Trail-making A time in seconds. Sust. Att. Hit: Sustained Attention Hit rate. Symb. Comp. Tot.: Symbolic number comparison total accuracy score. N = number of participants. Mean, SD, SE and lower and upper bounds of 95% confidence intervals are shown

	Math	WIAT Reading	Dot Matrix	vsWM	Phon. Dec.	Spatial Or. (%)	Trail-A time (sec)	WISC Vocabulary	Block Design	Raven	verbal WM	Digit Recall	Sust. Att. Hit (%)	Symb. Comp. Tot. (%)
*N*	98	98	98	98	98	98	98	98	98	98	98	98	94	97
Mean	97.9	107.5	100.6	111.2	101.8	29.6%	45.5	10.6	10.1	107.8	99.7	101.8	69.5%	91.7%
*SD*	14.5	15.3	15.8	12.7	9.6	21.1%	12.8	2.8	3.8	14.4	14.0	16.1	13.6%	6.7%
*SE*	4.6	4.9	5.0	4.0	3.0	6.7%	4.0	0.9	1.2	4.6	4.2	4.9	3.5%	2.0%
Ci:2.5%	95.0	104.4	97.4	108.6	99.9	25.4%	43.0	10.0	9.3	104.9	96.9	98.5	66.8%	90.4%
Ci:97.5%	100.8	110.6	103.8	113.7	103.8	33.8%	48.1	11.1	10.9	110.6	102.5	105.0	72.3%	93.1%

### Bootstrap correlations

**Figure 1:**
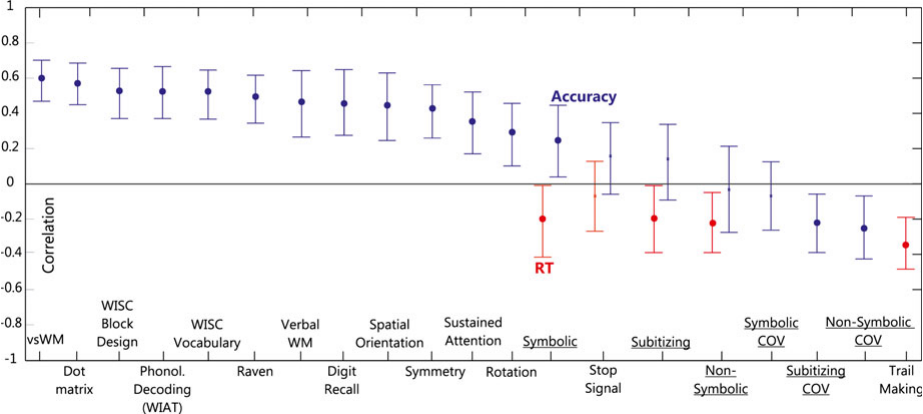
95% bootstrap confidence intervals for zero-order correlations. Significant correlations are marked by big bold dots. Non-significant correlations are marked by small dots. Blue lines show confidence intervals for test scores and accuracy. Red lines show confidence intervals for median RT. Symbolic/ Non-symbolic: symbolic and non-symbolic comparison tasks.

In order to assess the robustness of correlations, a bootstrap procedure determined empirical 95% confidence intervals for zero-order correlations. Figure1 shows empirical 95% bootstrap confidence intervals for zero-order correlations between math and those variables which showed significant partial and/or zero-order correlations with math. Correlations for all number sense measures are also shown. All measures which showed significant partial correlations with math showed robust zero-order correlation with math except the stop signal task. Descriptive statistics for measures showing significant partial and bootstrap correlations with math are shown in Table2. In addition to the above, while only symbolic number comparison task total accuracy showed significant partial correlation with math, some other number sense measures also showed zero-order bootstrap correlations with math (see Figure1). Descriptive statistics for number sense measures are provided in Table3.

**Table 3:** Descriptive statistics for number sense measures. Non-Symb. / Symbolic / Subitizing Total: Total accuracy in the non-symbolic task, symbolic and subitizing tasks. COV: Coefficient of Variation in the three above tasks. N = number of participants. Mean, SD, SE and lower and upper bounds of 95% confidence intervals are shown

	Non-Symb Total (%)	Symbolic Total (%)	Subitizing Total (%)	Non-Symb COV (RT)	Symbolic COV (RT)	Subitizing COV (RT)
*N*	97	97	97	97	97	97
Mean	90.6%	87.2%	86.7%	0.34	0.29	0.24
*SD*	21.2%	21.1%	21.2%	0.15	0.10	0.13
*SE*	8.7%	8.6%	8.6%	0.06	0.04	0.05
Ci:2.5%	86.4%	83.0%	82.5%	0.31	0.27	0.21
Ci:97.5%	94.8%	91.4%	90.9%	0.37	0.31	0.26

### Regression modelling

**Figure 2:**
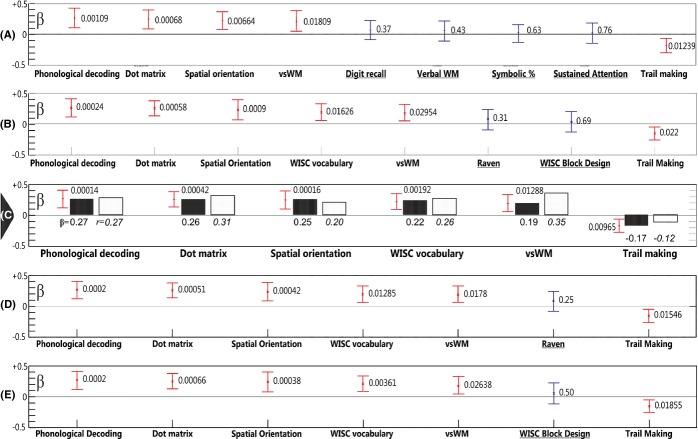
Model parameters. Y axes show β and r values for individual predictors. Mean β values (x) and 95% bootstrap confidence intervals are shown for individual predictor regression β values. The permutation test p value is shown next to confidence interval bars. Significant predictors are marked by red bars, non-significant predictors are marked by blue bars. Panels A–E show various models explained in the text. Panel C shows the best model. In Panel C the dark bars represent β values (the number below is the β value), the light bars represent zero-order r values (the number below is the r value). The comparison of β values and correlation values suggest that the model was stable. Permutation tests showed that all overall models were significant at the p < .00001 level.

Regression outcomes including bootstrap confidence intervals for β values and permutation testing results are summarized in Figure2. Supplementary Table3 shows initial steps of setting up a model and Table4 shows refining the model. Because of the lack of robust bootstrap correlation with math, the Stop Signal task was omitted from the initial potential predictor pool. First, the nine main variables of interest (which showed significant partial and bootstrap correlations with math) were entered into the regression (Supplementary Table3; M1). The regression explained 65% of variance. Dot matrix, visual WM, Phonological Decoding, Spatial Orientation and Trail-making were significant predictors. In contrast, verbal WM, Digit Recall, Symbolic number comparison accuracy and Sustained Attention were not significant predictors. The initial model is also shown in Figure2A with bootstrap confidence intervals and permutation testing outcomes which confirmed parametric statistics results.

**Table 4:** Optimizing regression models for predicting math performance (N =98 for all models; 51 girls). vs. WM: visual memory. Phon. Dec.: Phonological Decoding. Spatial Orient.: Spatial Orientation. Significant p values are marked in red

Model	*R*^2^/*F* − *p*	β/p	Dot Matrix	vsWM	Phon. Dec.	Spatial Orient.	Trail-Making	WISC Vocab	Block Design	Raven
M1	0.64/33.01	β	0.24	0.26	0.33	0.26	−0.19	–	–	–
	<0.0001	*p*	.0017	.0009	.0000	.0001	.0055	–	–	–
M2	0.68/24.01	β	0.25	0.17	0.26	0.23	−0.15	0.18	0.03	0.08
	<0.0001	*p*	.0008	.0289	.0002	.0008	.0216	.0156	.6883	.3051
M3^*^	0.68/31.93	β	0.26	0.19	0.27	0.25	−0.17	0.22	–	–
	<0.0001	*p*	.0005	.0130	.0001	.0001	.0090	.0020	–	–
M4	0.68/27.68	β	0.25	0.18	0.26	0.23	−0.16	0.19	–	0.09
	<0.0001	*p*	.0005	.0177	.0002	.0004	.0144	.0129	–	.2462
M5	0.68/27.27	β	0.25	0.17	0.27	0.24	−0.16	0.21	0.05	–
	<0.0001	*p*	.0008	.0269	.0001	.0004	.0176	.0038	.5053	–

In order to establish the potential individual importance of the above non-significant predictors, each of the non-significant predictors was entered into regressions one-by-one along with the five above-established significant predictors (Dot matrix, visual WM, Reading, Spatial Orientation and Trail-making). That is, in each step only a single, sixth variable was added to the above five significant predictors. Results are shown in M2–5 in Supplementary Table3. In each of these analyses the amount of explained variance ranged between 63 and 65%. β values related to each added variable remained non-significant. In contrast, all originally significant β values associated with the above five significant predictors remained significant. An additional regression added the Stop Signal Task accuracy to the five significant predictors (M6 in Supplementary Table3). The related β value was non-significant while Beta values related to the five originally significant predictors remained significant. Hence, the five non-significant variables (verbal WM, Digit Recall, Symbolic number comparison accuracy, Sustained Attention, Stop Signal Task) were omitted from all further analyses. Two additional separate regressions added Symmetry and Mental Rotation scores to the five significant predictors because these showed significant zero-order (but not partial) correlations with math (M7–8 in Supplementary Table3). The related β values were non-significant while β values related to the five originally significant predictors remained significant. Hence, these variables were also omitted from further analyses.

Table4; M1 shows the model with only the above five significant predictors. The following steps examined the relevance of general IQ variables for the above model. It is important to emphasize that the order of entry is not important when using simultaneous regressions. First, the three main IQ scores (WISC Vocabulary, Raven and Block Design) were added to the five remaining variables (Table4; M2 and Figure2B). Only WISC Vocabulary was a significant predictor. Hence, Raven and WISC Block Design were omitted from the following model (Table4; M3 and Figure2C). Raven (Table4; M4 and Figure2D) and WISC Block Design (Table4; M5 and Figure2E) remained non-significant even when they were entered into the regression without the other non-verbal IQ variable. In addition, the *R*^2^ value of the whole model did not change when adding either Raven or WISC Block Design. Further, the significance of significant predictors did not change when Raven and WISC Block design were added to the model. That is, adding IQ variables did not affect other variables’ predictive effect. Hence, the model in Table4; M3 and Figure2C was considered the best solution because this explained the highest amount of variance with the least number of variables with significant β values. This selected best model (Table4; M3; with WICS Vocabulary added) was also compared to the starting model (Table4; M1) with a partial *F* test. The model fit was significantly better in the selected ‘best’ model than in the starting model (*F* = 10.14; *p* = .0019). Notably, in all of the above analyses parametric, permutation and bootstrap procedures produced exactly the same outcome. The best model was also tested with Gender as an additional dichotomous dummy coded variable. Gender was a non-significant predictor (β = −0.06; *p* = *ns* [0.35]). The bootstrap resampling provides a robust method for estimating confidence intervals for β values and determining *p* values. In addition, the best model conformed well to the assumptions of multiple linear regression and VIF values were low (see Supplementary Results and Supplementary Figure1).

### Testing the specificity of the model

The specificity of the model was tested by using the above best model (Figure2C) to predict reading rather than math performance. The dependent variable was the Hodder Group Reading Test which was not included in any of the predictors and showed a moderate (*r* = 0.57) zero-order correlation with the Phonological Decoding predictor variable. The model provided a good fit (*N* = 98; *R*^2^ = 0.39; *F* = 9.57; *p* < .0001). However, only Phonological Decoding (β = 0.424; *p* < .0001) and WISC Vocabulary (β = 0.23; *p* = .017) were significant individual predictors. Other variables showed negligible β values (0.005 ≤ β ≤ 0.08; 0.444 ≤ *p* ≤ .948). The model fit remained unchanged when only Phonological Decoding (β = 0.462; *p* < .0001) and WISC Vocabulary (β = 0.267; *p* < .0001) were used as predictors (*N* = 98; *R*^2^ = 0.39; *F* = 30.10; *p* < .0001). The model fit improved slightly when Sustained Attention (which showed a significant partial correlation with the Hodder Group Reading Test) was also added to the model (*N* = 95; *R*^2^ = 0.43; *F* = 22.85; *p* < .0001. Phonological Decoding: β = 0.461; *p* < .0001. WISC Vocabulary: β = 0.207; *p* < .0001. Sustained Attention: β = 0.15; *p* = .095). The above demonstrates that the best math model was highly specific to mathematics and predicting reading performance would require a substantially different model. VIF values for all variables used ranged between 1.22 and 1.93.

### Forced prediction of math from number sense variables

**Figure 3:**
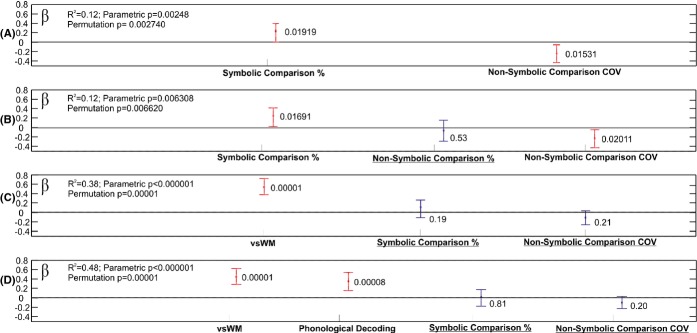
Model parameters for testing number sense variables. Y axes show β and r values for individual predictors. Panels A–D show various models explained in the text. Overall model R^2^ and parametric and permutation testing p values are shown on the left of each panel. For individual predictors 95% bootstrap confidence intervals for regression β values are shown. Permutation p values for individual predictor β values are shown next to confidence intervals.

While none of the number sense measures proved a reliable predictor of math performance it was nevertheless theoretically important to clarify their relation to math. The analysis started from number sense measures. Final results are summarized in Figure3. Only regression models based solely on total accuracy data and RT COV identified significant predictors. Hence, the results below relate to these measures. Results are summarized in Supplementary Table4. First, all number sense measures showing robust zero-order correlations with math were entered into the regression (Supplementary Table4; M1). Only total symbolic comparison accuracy and Non-symbolic comparison COV showed significant connection to math. The *R*^2^ value was much smaller than in the above analyses with additional variables. Results remained practically unchanged with only three number sense variables in regression. M2 tested subitizing COV while M3 tested total non-symbolic task accuracy. M4 only included the two significant number sense measures. M5–10 successively added one of the non-number sense variables identified in the best model predicting math (Supplementary Table4; M3 and Figure2C). M11 added two of the above variables. M12 added all of the significant variables from the best model. In order to assess the robustness of models and predictors, bootstrap and permutation analyses were run with 100,000 permutations for some of the above models (see results in Figure3). Non-symbolic comparison COV invariably became non-significant when there was even a sole additional visuo-spatial predictor variable (visuo-spatial WM; Dot Matrix; Spatial Orientation) in the regression equation. Total symbolic comparison accuracy remained significant as long as visuo-spatial WM and reading were not added to the regression. None of the number sense measures proved to be reliable when there were more than two non-number sense predictors in the model. In contrast, other variables were extremely robust.

### Predicting number sense variables

While it was not the main objective of the current study, it is theoretically important to identify potential predictors of number sense variables. Details are described in the Supplementary Results. In summary, Sustained Attention emerged as the most robust predictor of number sense variables and Phonological Decoding and the Dot Matrix task were also predictors in some analyses.

## Discussion

We contrasted the predictive power of various cognitive variables and several variables associated with a proposed number sense in a robust, permutation testing based framework in 9-year-old children with normal reading. Verbal intelligence and phonological ability, visual STM and WM, spatial ability and trail-making task performance emerged as robust predictors of mathematical achievement. Non-verbal IQ measures were non-significant predictors in the above model and the model was highly specific to predicting mathematical performance. None of the number sense measures proved to be serious predictors of mathematical achievement. We propose an executive memory function centric model of mathematical expertise.

### A mathematical processing network

Our data are consistent with an extended number processing network (Supekar *et al*., 2009; Bressler & Menon, 2010; Menon, 2010; Fias *et al*., 2013; Goswami & Szűcs, 2011; Dowker, 2005). Based on our measures, we conclude that important processing nodes of this network are phonological decoding, verbal knowledge, visuo-spatial memory, spatial ability and general executive functioning measured by the trail-making task. This is in line with several studies cited in the Introduction. WISC Block design and Raven scores were non-significant predictors when tested with our best model. Thus, the well specified spatial components of our model (both visuo-spatial STM/WM and spatial orientation) better explained variance than the functionally less clearly defined Raven and/or WISC Block design scores. Only spatial orientation but not symmetry score showed a relationship with math. This suggests that more active spatial processing components are likely to be related to math performance, probably because these are more related to spatially based mental operations required by mathematics.

We found no strong relations between mathematical achievement and verbal STM/WM, simple detection task RT, sustained attention, stop signal task performance, a symmetry task, a mental rotation task, finger knowledge and line bisection performance. Our study included children with at least average reading skill. Hence, finding strong relations between visuo-spatial STM/WM measures and mathematics but not with verbal STM/WM measures is in line with recent studies controlling for poor reading skill (Schuchardt *et al*., 2008; see also Van der Sluis *et al*., 2005; Szűcs *et al*., 2013a). These studies together with our data and with studies reporting poor verbal WM in children with reading comprehension problems (Helland & Asbjornsen, 2004; Hecht *et al*., 2001; Schuchardt *et al*., 2008; Pimperton & Nation, 2010) suggest that verbal STM/WM is primarily related to reading comprehension while visual STM/WM is more related to mathematical ability. With regard to this it is important that the optimal model predicting reading achievement was substantially different from the model predicting mathematical achievement with only verbal IQ and phonological decoding predicting reading performance significantly. Hence, while there is substantial shared variance between reading and mathematical performance, our data suggest that this shared variance relies on verbal IQ and phonological decoding which are equally important for both reading and mathematics. In contrast, visual STM/WM, spatial ability and trail-making performance were only related to mathematics but not to reading comprehension.

### Number sense related variables

Recent cognitive neuroscience research on numerical cognition has shifted the focus of research to a putative magnitude representation supposedly core to mathematical function. Strong claims have been made about the importance of this proposed number sense for mathematical development and performance in children at various ages and in adults (Halberda *et al*., 2008; Piazza *et al*., 2010; Halberda, Ly, Wilmer, Naiman & Germine, 2012). Our data do not support these claims and suggest that number sense measures are in fact not directly related to mathematical performance if other more robust predictors are taken into account (see also Mix, Levine & Huttenlocher, 1997; Mix, Huttenlocher & Levine, 2002; Rouselle & Noël, 2007; Soltész *et al*., 2007; Holloway & Ansari, 2008; Schneider, Grabner & Paetsch, 2009; Landerl & Kolle, 2009; Kovas, Giampietro, Viding, Ng, Brammer, Barker, Happé & Plomin, 2009; Soltész *et al*., 2010; Szücs *et al*., 2013a). That is, our data in effect falsify claims about the importance of a modular number sense for mathematical performance, at least in 9-year-old children. Our conclusions are in line with recent papers which found that non-symbolic magnitude discrimination performance did not predict mathematical performance when inhibitory control was taken into account (Fuhs & McNeil, 2013; Gilmore, Attridge, Clayton, Cragg, Johnson, Marlow, Simms & Inglis, 2013).

Here, number sense measures produced weak but significant effect sizes when entered into regressions alone. This is compatible with the results of some studies claiming support for number sense. Halberda *et al*. (2012) reported a correlation of *r* = −0.16 between one number sense measure and standardized mathematical performance in 458 individuals while not controlling for other variables. This correlation is even weaker than the correlation values we measured here. However, our analyses demonstrate that number sense variables cease to be significant predictors of mathematics as soon as more robust predictors are added to models (also note that regression assumptions do not seem to have been tested in number sense studies). This suggests that several reports of a predictive relationship between number sense and mathematical performance may have been spurious and were due to the fact that other better predictors of mathematical performance were not entered into regressions and/or were not measured/used in a valid way. An additional point regarding the validity of non-symbolic number sense measures is that these measures are inherently confounded by visual stimulus parameters which may distort results (see Szűcs, Nobes, Devine, Gabriel & Gebuis, 2013b; Gebuis & Reynvoet, 2012; Clearfield & Mix, 1999; Mix *et al*., 1997).

In our data both non-symbolic and symbolic number sense variables were related to sustained attention. This makes sense because in these tasks children perform a tedious and sometimes difficult task with relatively quick presentation times for a sustained period. A relationship between non-symbolic magnitude comparison performance and selective attention may also explain intraparietal sulcus (IPS) activity in magnitude comparison tasks as IPS activity is known to be modulated by attention (Coull & Frith, 1998; Vandenberghe, Molenberghs & Gillebert, 2012; Santangelo & Macaluso, 2013; Davranche, Nazarian, Vidal & Coull, 2011; Culham & Kanwisher, 2001). In addition, non-symbolic comparison performance seemed connected to visual STM (dot matrix task). Further, both non-symbolic and symbolic comparison tasks ceased to predict arithmetic when visuo-spatial variables were added to regressions (visual STM/WM and spatial orientation scores). Provided that both comparison tasks require the involvement of memory processes, particularly the non-symbolic comparison task which may rely on visual memory (e.g. Xenidou-Dervou, Van Lieshout & van der Schoot, 2013), our data are also in agreement with studies which linked visual memory related IPS activity to mathematical performance (Rotzer, Loenneker, Kucian, Martin, Klaver & von Aster, 2009; Dumontheil & Klingberg, 2011).

Symbolic number comparison accuracy was more connected to mathematical scores than non-symbolic comparison accuracy which is in line with several studies (Nosworthy, Bugden, Archibald, Evans & Ansari, 2013; Holloway & Ansari, 2009; Noël & Rouselle, 2011; De Smedt & Gilmore, 2011). However, our data also suggest that symbolic number comparison performance is probably more directly related to reading performance and phonological decoding than to arithmetic performance *per se*. This would suggest that this task may be more important as a measure of the developmental level of symbolic labelling/understanding than a measure of symbol to magnitude representation connections. It is important to point out that although we emphasize the role of domain-general variables we do not deny the relevance of mathematics subject-specific knowledge for mathematical understanding. It is obvious that such knowledge is important together with more general cognitive abilities (see Jordan, Hansen, Fuchs, Siegler, Gersten & Micklos, 2013; Geary, Hoard, Nugent & Bailey, 2013; Träff, 2013). What we show in relation to proposed mathematics domain specific variables is that a proposed number sense (a non-symbolic magnitude representation or approximate number system) is not relevant (or it has negligible relevance) for mathematical performance in 9-year-old children.

### An executive memory function centric model of mathematical processing

**Figure 4:**
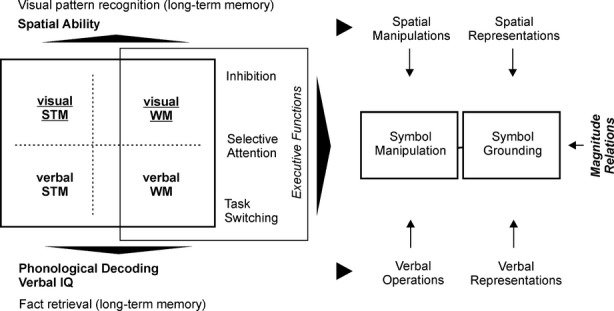
A preliminary, executive memory function centric model of mathematical expertise. The variables tested in this study are bold. Working memory function relies on specific executive functions and is core to mathematical processing. Visuo-spatial STM/WM (underlined) seems more directly related to mathematics while verbal STM/WM contributes to both reading and mathematics. Spatial abilities most likely rely on visuo-spatial STM/WM capacity. Phonological processing and verbal IQ most likely rely on verbal STM/WM capacity. Numerous arithmetic facts (e.g. multiplication tables and formulas) are retrieved from long-term verbal memory but long-term memory based visual pattern recognition is probably also important (e.g. at more advanced stages when quickly recognizing identities like [a − b]^2^ = a^2^ − 2ab + b^2^). The network serves mathematical symbol processing and symbol manipulation. Symbols need to be grounded initially by linking them e.g. to magnitudes and count words. However, our study suggests that the level of this symbol grounding is not relevant to age-appropriate mathematical performance after the initial grounding process.

Besides modular theories not being developmentally very feasible (Karmiloff-Smith, 1995), a network view of mathematical ability is highly plausible because it fits very well the heterogeneity of mathematical weaknesses (see Rubinsten & Henik, 2009). Figure4 outlines a preliminary ‘executive memory function centric’ model of mathematical processing. The focus on executive memory processes relies on the large volume of studies confirming the importance of working memory function for mathematics (Raghubar *et al*., 2010, for review) and on studies of developmental dyscalculia reporting working memory function disruption (Szűcs *et al*., 2013a, for review).

Following Miyake *et al*. (2000), we assume that executive functions (attentional focus shifting related to selective attention; information updating and monitoring; inhibition of irrelevant information) form the core of (central) executive memory processes. These executive processes are necessary to control the workflow of activities taking place in working memory, for example selecting stimuli for momentary operations. Most studies in numerical cognition have relied on the initial formulation of Baddeley's WM model (1986) assuming domain-general central executive (CE) function. However, the working memory literature (Shah & Miyake, 1996; Miyake *et al*., 2000; Jarvis & Gathercole, 2003) and numerical development studies (Schuchardt *et al*., 2008; Van der Sluis *et al*., 2005; Szűcs *et al*., 2013a) suggest that verbal and visual WM function can dissociate. Verbal and visual memory processes most likely support verbal and spatial operations. These operations serve abstract symbol manipulation which is the most important characteristic of human mathematics. Recent studies suggest that visual memory processes may be more directly important for mathematical processing than verbal memory (current study; Schuchardt *et al*., 2008; see also Van der Sluis *et al*., 2005; Szűcs *et al*., 2013a). During the first school year (Rubinsten, Henik, Berger & Shahar-Shalev, 2002) symbols are linked to their referents, like magnitudes (Ansari, 2008) and counting words. Our study suggests that the relevance of this link for later age-appropriate mathematical processing is limited.

The majority of pupils are likely to consider math a difficult subject (Brown, Brown & Bibby, 2006) and math is able to elicit specific anxiety from pupils (Ashcraft, 2002; Maloney & Beilock, 2012). Math being difficult and a somewhat special subject is a plausible introspection for the following reasons: First, abstraction (symbolizing problems) matures only by late childhood (Markovits & Lortie-Forgues, 2011). Second, abstract symbol manipulation is a very unnatural task for the human mind (Cohen, 1981; Oaksford & Chater, 1994). Third, we suggest math difficulties also relate to the extensively networked nature of processing requirements: Age-appropriate math probably taxes all the elements of a processing network to their maximum capacity virtually at the same time and coordinating elements of the network probably puts considerable burden on executive (memory) processes. Due to these high processing demands, math does not have any fail tolerance: a perfect computational result has to be achieved typically under time pressure and task difficulty is increasing all the time during schooling. Hence, the processing network must perform perfectly, at peak levels, under pressure and minor mistakes unrelated to the subject matter of math (like misremembering a sign) can lead to vastly incorrect results. Hence, any weakness at any nodes in the extended processing network, especially in executive functions coordinating network activities, can lead to catastrophic consequences in task solutions. The heterogeneity of mathematical weaknesses seems straightforward when considering the above: Due to high demand (task difficulty), any (minor) weaknesses of the extensive processing network may result in disrupted mathematical performance. In contrast, other school activities may be less demanding than mathematics and/or allow for more compensation by alternative processing nodes if one network node is weak. Hence, a problem with a particular processing node (e.g. weak visuo-spatial memory) can become very apparent in mathematics but may cause much less serious difficulties in other subjects.

Our study has some limitations. First, we tested an unusually large number of variables which did not allow us to test more than one age group. However, the predictor space of mathematics is probably changing throughout development (Karmiloff-Smith, 1995). For example, understanding magnitude relations of digits may be most important at the beginning of primary school when children are actually learning these relations (Rubinsten *et al*., 2002). Hence, symbolic magnitude comparison may be more related to mathematical ability earlier rather than later in education (e.g. Holloway & Ansari, 2009). Extensive cross-sectional and/or longitudinal studies are needed to map the changing developmental landscape and determine the age-appropriate relevance of variables. Second, mathematics consists of several major domains, e.g. arithmetic and geometry, and specific operations may rely on different nodes of a processing network. Hence, future studies could explore to what extent models based on different groups of nodes of a large processing network are specific to certain mathematical domains and/or operations. Third, while most of our measures were well-established standardized measures, the reliability of some measures, most importantly that of spatial orientation ability, needs to be established. Fourth, while we identify important nodes of a mathematical processing network, of course more work is needed before a formal computational model of network interactions between these nodes can be defined.

### Conclusions

We suggest an ‘executive memory function centric’ model of a mathematical processing network and in line with Fias *et al*. (2013) we suggest that research on mathematical development should shift towards identifying mechanisms of mental processes operating on (symbolic) representations, especially those of executive memory processes. Number sense variables had negligible explanatory power which seriously challenges the number sense theory. We suggest that previous studies measured spurious correlations between number sense variables and mathematical performance. Valid mapping of the extensive mathematical processing network requires testing significant variables in their extended psychological context rather than in isolation. Major questions are how much particular nodes of the mathematical network can be trained, whether one node can compensate for the weakness(es) of (an)other one(s), whether training network nodes in the context of mathematical tasks is more effective than training them on their own, and how exactly various processing nodes enable mathematical symbol manipulation.

## Supporting Information

Figure S1 Tests of regression assumptions for the best multiple linear regression model shown in Figure 2C.

Table S1 The dot number pairs for each ratio. **Table S2****.** Zero order and partial correlations. Test abbreviations: *HGRT*: Hodder Group Reading Test. *WB*: WISC Block Design. *WV*: WISC Vocabulary. *Rav*: Raven's CPM. *vsWM*: visual WM. *verbWM*: verbal WM. *Phon.Dec*: Phonological decoding. *Sp. Orient*: Spatial Orientation. *Sust.Att*: Sustained attention. *Non-symb. / Symb. / Subit*.: Non-symbolic comparison / symbolic comparison / Subitizing tasks; Tot: Total accuracy; COV: Coefficient of Variation. RT: Reaction Time. Significance levels: red: p<0.001; magenta: p<0.01; blue: p<0.05. **Table S3.** Initial regression models for predicting math performance. Spatial Or.: Spatial Orientation. Sus. Att.: Sustained Attention. Symb tot: Symbolic number comparison total accuracy. vsWM: visual memory. Significant p values are marked by red. **Table 4.** Regression models for predicting math performance from number sense variables (51 girls in all models). Significant p values are marked by red, marginally significant p values are marked by blue. Variance Inflation Factors (VIF) ranged between 1.20 - 2.01.
