# Supplementary material for: Cognitive components of a mathematical processing network in 9-year-old children
Source: Dev Sci. 2014 Feb 23;17(4):506–24. doi: 10.1111/desc.12144 (PMC4253132; doi:10.1111/desc.12144)
Supplement: Table S1 — The dot number pairs for each ratio. Table S2. Zero order and partial correlations. Test abbreviations: HGRT: Hodder Group Reading Test. WB: WISC Block Design. WV: WISC Vocabulary. Rav: Raven's CPM. vsWM: visual WM. verbWM: verbal WM. Phon.Dec: Phonological decoding. Sp. Orient: Spatial Orientation. Sust.Att: Sustained attention. Non-symb. / Symb. / Subit.: Non-symbolic comparison / symbolic comparison / Subitizing tasks; Tot: Total accuracy; COV: Coefficient of Variation. RT: Reaction Time. Significance levels: red: p<0.001; magenta: p<0.01; blue: p<0.05. Table S3. Initial regression models for predicting math performance. Spatial Or.: Spatial Orientation. Sus. Att.: Sustained Attention. Symb tot: Symbolic number comparison total accuracy. vsWM: visual memory. Significant p values are marked by red. Table 4. Regression models for predicting math performance from number sense variables (51 girls in all models). Significant p values are marked by red, marginally significant p values are marked by blue. Variance Inflation Factors (VIF) ranged between 1.20 - 2.01. [file desc0017-0506-SD2.docx]

**Supplementary tables**

**Supplementary Table 1:** The dot number pairs for each ratio.

| **1 : 2** | **3 : 5** | **2 : 3** |
| --- | --- | --- |
| 4 8 * | 6 10 * | 8 12 * |
| 6 12 ** | 9 15 ** | 12 18 **^/^° |
| 10 20 ° | 12 20 ° |  |

Table from Soltész et al. (2010). Ratios are in columns. * and ** indicate dot pairs which are the same numerical distance (the numerical distances are 4 and 6, respectively). The overall sum is almost equal for dot number pairs marked with ° (30, 32 and 30)

**Supplementary Table 2:** Zero order and partial correlations. Test abbreviations: *HGRT*: Hodder Group Reading Test. *WB*: WISC Block Design. *WV*: WISC Vocabulary. *Rav*: Raven's CPM. *vsWM*: visual WM. *verbWM*: verbal WM. *Phon.Dec*: Phonological decoding. *Sp. Orient*: Spatial Orientation. *Sust.Att*: Sustained attention. *Non-symb. / Symb. / Subit*.: Non-symbolic comparison / symbolic comparison / Subitizing tasks; Tot: Total accuracy; COV: Coefficient of Variation. RT: Reaction Time. Significance levels: red: p<0.001; magenta: p<0.01; blue: p<0.05.

|  |  | **Math** | | **HGRT** | | **WB** | **WV** | **Rav** | **Dot Matrix** | | **vsWM** | | **verbWM** | | **Phon.Dec.** | | **Sp. Orient.** | | **Trail-A Time** | | **Stop Signal** | |
| --- | --- | --- | --- | --- | --- | --- | --- | --- | --- | --- | --- | --- | --- | --- | --- | --- | --- | --- | --- | --- | --- | --- |
|  | order of correlation> | Zero | Partial | Zero | Partial | Zero | Zero | Zero | Zero | Partial | Zero | Partial | Zero | Partial | Zero | Partial | Zero | Partial | Zero | Partial | Zero | Partial |
|  | **HGRT** | 0.37 |  |  |  |  |  |  |  |  |  |  |  |  |  |  |  |  |  |  |  |  |
|  | **WISC Block (WB)** | **0.53** | -- | **0.26** | -- |  |  |  |  |  |  |  |  |  |  |  |  |  |  |  |  |  |
|  | **WISC Vocab (WV)** | **0.53** | -- | **0.46** | -- | **0.39** |  |  |  |  |  |  |  |  |  |  |  |  |  |  |  |  |
|  | **Raven (Rav)** | **0.5** | -- | **0.28** | -- | **0.48** | **0.5** |  |  |  |  |  |  |  |  |  |  |  |  |  |  |  |
|  | **Dot Matrix** | **0.57** | **0.48** | **0.28** | 0.19 | **0.42** | **0.2** | **0.26** |  |  |  |  |  |  |  |  |  |  |  |  |  |  |
|  | **vsWM** | **0.6** | **0.38** | **0.37** | 0.19 | **0.51** | **0.42** | **0.37** | **0.49** | **0.35** |  |  |  |  |  |  |  |  |  |  |  |  |
|  | **Verbal WM** | **0.47** | **0.24** | **0.36** | 0.16 | **0.3** | **0.51** | **0.3** | **0.43** | **0.37** | **0.51** | **0.37** |  |  |  |  |  |  |  |  |  |  |
|  | **Phonol. Decoding** | **0.53** | **0.39** | **0.57** | **0.47** | **0.23** | **0.41** | **0.31** | **0.27** | 0.19 | **0.36** | **0.21** | **0.28** | 0.08 |  |  |  |  |  |  |  |  |
|  | **Spatial Orientation** | **0.45** | **0.32** | 0.13 | 0.02 | **0.37** | 0.17 | **0.3** | **0.23** | 0.09 | **0.28** | 0.1 | 0.16 | 0.05 | 0.11 | 0 |  |  |  |  |  |  |
|  | **Trail-A time** | **-0.35** | **-0.22** | -0.05 | 0.04 | **-0.35** | -0.14 | **-0.22** | **-0.29** | -0.18 | -0.19 | -0.01 | **-0.21** | -0.13 | -0.03 | 0.07 | -0.14 | 0 |  |  |  |  |
|  | **Stop Signal** | 0.16 | **0.21** | 0.09 | 0.05 | -0.1 | 0.18 | -0.11 | 0.19 | **0.27** | **0.23** | **0.3** | **0.25** | **0.23** | 0.16 | 0.12 | -0.02 | 0.04 | -0.2 | **-0.27** |  |  |
|  | **Sustained Attention** | **0.36** | **0.21** | **0.4** | **0.28** | 0.13 | **0.39** | **0.27** | 0.1 | 0.03 | **0.31** | 0.2 | **0.26** | 0.07 | **0.37** | **0.25** | **0.29** | **0.26** | -0.13 | -0.08 | 0.15 | 0.11 |
| **RT** | **Non-Symb Total** | **-0.25** | -0.14 | -0.02 | 0.12 | -0.18 | **-0.24** | -0.1 | -0.16 | -0.09 | -0.13 | 0 | -0.11 | 0.02 | -0.04 | 0.06 | **-0.22** | -0.18 | 0.18 | 0.13 | -0.08 | -0.05 |
|  | **Symbolic Total** | **-0.21** | -0.1 | 0.17 | 0.04 | -0.11 | **-0.27** | -0.13 | **-0.29** | **-0.26** | -0.14 | -0.04 | **-0.25** | -0.14 | -0.1 | 0.01 | **-0.24** | **-0.22** | 0.14 | 0.11 | -0.05 | -0.01 |
|  | **Subit Total** | **-0.25** | -0.08 | 0.08 | 0.05 | **-0.32** | -0.17 | **-0.23** | **-0.21** | -0.09 | -0.19 | -0.02 | -0.16 | -0.06 | -0.07 | 0.03 | **-0.26** | -0.15 | 0.1 | -0.02 | 0.13 | 0.1 |
| **Accuracy** | **Non-Symbolic** | -0.03 | -0.02 | -0.01 | 0 | -0.08 | -0.05 | 0.11 | 0 | 0.02 | 0.13 | 0.2 | -0.02 | 0.01 | 0.03 | 0.04 | 0.05 | 0.05 | 0.01 | -0.01 | 0.14 | 0.2 |
|  | **Symbolic** | **0.25** | **0.28** | -0.08 | 0.15 | -0.05 | 0.11 | 0.15 | 0.09 | 0.11 | **0.24** | **0.28** | 0.13 | 0.1 | **0.33** | **0.32** | 0.05 | 0.05 | -0.04 | -0.06 | **0.34** | **0.36** |
|  | **Subitizing** | 0.14 | 0.14 | -0.06 | 0.1 | 0.14 | -0.05 | 0.03 | 0.11 | 0.07 | 0.1 | 0.07 | -0.07 | -0.08 | 0.04 | 0.05 | 0.15 | 0.12 | -0.16 | -0.12 | -0.04 | -0.02 |
|  | **Non-Symb COV** | **-0.26** | -0.13 | -0.08 | 0.02 | **-0.25** | -0.19 | -0.15 | **-0.36** | **-0.29** | **-0.26** | -0.14 | -0.14 | -0.03 | -0.11 | -0.02 | **-0.24** | -0.17 | 0.09 | 0 | -0.13 | -0.13 |
|  | **Symbolic COV** | -0.08 | -0.03 | -0.05 | 0.02 | 0 | -0.19 | 0 | -0.15 | -0.17 | -0.16 | -0.15 | -0.05 | 0.04 | -0.15 | -0.1 | -0.18 | **-0.21** | -0.02 | -0.02 | **-0.21** | -0.16 |
|  | **Subitizing COV** | **-0.22** | -0.09 | 0 | 0.09 | **-0.24** | -0.14 | -0.18 | -0.13 | -0.03 | -0.12 | 0.03 | -0.09 | 0 | -0.1 | -0.03 | -0.19 | -0.1 | **0.2** | 0.13 | -0.03 | -0.06 |

**Suppl. Table 2: first part**

|  |  | **Sust. Att** | | **Non-Symb Tot** | | **Symb Tot** | | **Subit Tot** | | **NS COV** | | **Symb COV** | |
| --- | --- | --- | --- | --- | --- | --- | --- | --- | --- | --- | --- | --- | --- |
|  | order of correl. > | Zero | Partial | Zero | Partial | Zero | Partial | Zero | Partial | Zero | Partial | Zero | Partial |
|  | **HGRT** |  |  |  |  |  |  |  |  |  |  |  |  |
|  | **WISC Block (WB)** |  |  |  |  |  |  |  |  |  |  |  |  |
|  | **WISC Vocab (WV)** |  |  |  |  |  |  |  |  |  |  |  |  |
|  | **Raven (Ra)** |  |  |  |  |  |  |  |  |  |  |  |  |
|  | **Dot Matrix** |  |  |  |  |  |  |  |  |  |  |  |  |
|  | **vsWM** |  |  |  |  |  |  |  |  |  |  |  |  |
|  | **rbWM** |  |  |  |  |  |  |  |  |  |  |  |  |
|  | **Phonol. Decoding** |  |  |  |  |  |  |  |  |  |  |  |  |
|  | **Spatial Orient.** |  |  |  |  |  |  |  |  |  |  |  |  |
|  | **Trail making** |  |  |  |  |  |  |  |  |  |  |  |  |
|  | **Stop Signal** |  |  |  |  |  |  |  |  |  |  |  |  |
|  | **Sustained Att.** |  |  |  |  |  |  |  |  |  |  |  |  |
| **RT** | **Non-Symb Total** | **-0.3** | **-0.24** |  |  |  |  |  |  |  |  |  |  |
|  | **Symbolic Total** | **-0.42** | **-0.35** | **0.59** | **0.56** |  |  |  |  |  |  |  |  |
|  | **Subit Total** | **-0.37** | **-0.34** | **0.44** | **0.41** | **0.37** | **0.35** |  |  |  |  |  |  |
| **Accuracy** | **Non-Symbolic** | 0 | 0 |  |  |  |  |  |  |  |  |  |  |
|  | **Symbolic** | **0.43** | **0.42** | **0.24** | **0.22** |  |  |  |  |  |  |  |  |
|  | **Subitizing** | 0.06 | 0.11 | 0.1 | 0.11 | 0.15 | 0.17 |  |  |  |  |  |  |
|  | **Non-Symb COV** | **-0.21** | -0.15 | 0.11 | 0.1 | -0.08 | -0.08 | -0.18 | -0.17 |  |  |  |  |
|  | **Symbolic COV** | **-0.34** | **-0.31** | **0.28** | **0.27** | -0.14 | -0.13 | -0.08 | -0.11 | **0.45** | **0.46** |  |  |
|  | **Subitizing COV** | **-0.29** | **-0.27** | -0.04 | -0.05 | -0.04 | -0.04 | -0.14 | -0.12 | **0.33** | **0.28** | 0.18 | 0.19 |

**Table 2: second part (on the right of the first part)**

**Supplementary Table 3**

Initial regression models for predicting math performance. Spatial Or.: Spatial Orientation. Sus. Att.: Sustained Attention. Symb tot: Symbolic number comparison total accuracy. vsWM: visual memory. Significant p values are marked by red.

| **Model** | **N** | **R^2^/F - p** | **β/p** | **Dot Matrix** | **vsWM** | **Phonology** | **Spatial Or.** | **Trail Making** | **verbal WM** | **Sus Att** | **Symb tot** | **Digit Recall** |
| --- | --- | --- | --- | --- | --- | --- | --- | --- | --- | --- | --- | --- |
| **M1** | 93 | 65/17.30 | β | 0.24 | 0.20 | 0.27 | 0.26 | -0.18 | 0.08 | 0.07 | 0.06 | 0.07 |
|  |  | <0.0001 | p | 0.0046 | 0.0255 | 0.0011 | 0.0006 | 0.0116 | 0.3742 | 0.4196 | 0.4061 | 0.3616 |
|  |  |  |  | **Dot Matrix** | **vsWM** | **Phonology** | **Spatial Or.** | **Trail Making** | **verbal WM** |  |  |  |
| **M2** | 98 | 65/27.76 | β | 0.22 | 0.23 | 0.33 | 0.26 | -0.18 | 0.08 |  |  |  |
|  |  | <0.0001 | p | 0.0039 | 0.0055 | 0.0000 | 0.0001 | 0.0075 | 0.2818 |  |  |  |
|  |  |  |  | **Dot Matrix** | **vsWM** | **Phonology** | **Spatial Or.** | **Trail Making** | **Sus Att** |  |  |  |
| **M3** | 98 | 65/27.78 | β | 0.23 | 0.24 | 0.31 | 0.25 | -0.19 | 0.08 |  |  |  |
|  |  | <0.0001 | p | 0.0022 | 0.0024 | 0.0000 | 0.0002 | 0.0052 | 0.2747 |  |  |  |
|  |  |  |  | **Dot Matrix** | **vsWM** | **Phonology** | **Spatial Or.** | **Trail Making** | **Symb tot** |  |  |  |
| **M4** | 94 | 64/26.07 | β | 0.24 | 0.25 | 0.31 | 0.26 | -0.18 | 0.04 |  |  |  |
|  |  | <0.0001 | p | 0.0021 | 0.0018 | 0.0000 | 0.0004 | 0.0082 | 0.5905 |  |  |  |
|  |  |  |  | **Dot Matrix** | **vsWM** | **Phonology** | **Spatial Or.** | **Trail Making** | **Digit Recall** |  |  |  |
| **M5** | 97 | 64/27.05 | β | 0.24 | 0.25 | 0.33 | 0.26 | -0.19 | 0.02 |  |  |  |
|  |  | <0.0001 | p | 0.0024 | 0.0014 | 0.0000 | 0.0002 | 0.0060 | 0.8062 |  |  |  |
|  |  |  |  | **Dot Matrix** | **vsWM** | **Phonology** | **Spatial Or.** | **Trail Making** | **Stop Signal** |  |  |  |
| **M6** | 96 | 63/25.12 | β | 0.26 | 0.26 | 0.29 | 0.26 | -0.20 | -0.03 |  |  |  |
|  |  | <0.0001 | p | 0.0011 | 0.0011 | 0.0001 | 0.0003 | 0.0044 | 0.6316 |  |  |  |
|  |  |  |  | **Dot Matrix** | **vsWM** | **Phonology** | **Spatial Or.** | **Trail Making** | **Symmetry** |  |  |  |
| **M7** | 98 | 64/27.34 | β | 0.24 | 0.25 | 0.33 | 0.25 | -0.18 | 0.04 |  |  |  |
|  |  | <0.0001 | p | 0.0018 | 0.0014 | 0.0000 | 0.0007 | 0.0119 | 0.6041 |  |  |  |
|  |  |  |  | **Dot Matrix** | **vsWM** | **Phonology** | **Spatial Or.** | **Trail Making** | **Rotation** |  |  |  |
| **M8** | 98 | 64/27.24 | β | 0.24 | 0.26 | 0.34 | 0.27 | -0.19 | -0.02 |  |  |  |
|  |  | <0.0001 | p | 0.0017 | 0.0010 | 0.0000 | 0.0002 | 0.0076 | 0.7970 |  |  |  |

**Supplementary Table 4**

Regression models for predicting math performance from number sense variables (51 girls in all models). Significant p values are marked by red, marginally significant p values are marked by blue. Variance Inflation Factors (VIF) ranged between 1.20 - 2.01.

| Model | **N** |  | **R^2^/F** | **β** | **Symbolic Total %** | **Subitizing 1-3 Total%** | **Non-Symb COV** | **Symbolic COV** | **Subitizing 1-3 COV** |  |  |  |
| --- | --- | --- | --- | --- | --- | --- | --- | --- | --- | --- | --- | --- |
| M1 | 95 | **R^2^/F:** | 0.15/3.16 | **β** | 0.24 | -0.09 | -0.23 | 0.11 | -0.17 |  |  |  |
|  |  | **p=** | 0.0113 | **p** | 0.0159 | 0.3562 | 0.0463 | 0.3083 | 0.1155 |  |  |  |
|  |  |  |  |  | **Symbolic Total %** | **Non-Symb COV** | **Subitizing 1-3 COV** |  |  |  |  |  |
| M2 | 95 | **R^2^/F:** | 0.13/4.71 | **β** | 0.23 | -0.19 | -0.14 |  |  |  |  |  |
|  |  | **p=** | 0.0042 | **p** | 0.0219 | 0.0713 | 0.1655 |  |  |  |  |  |
|  |  |  |  |  | **Symbolic Total %** | **Non-Symb COV** | **Non-Symb Total** |  |  |  |  |  |
| M3 | 97 | **R^2^/F:** | 0.12/4.37 | **β** | 0.25 | -0.23 | -0.06 |  |  |  |  |  |
|  |  | **p=** | 0.0063 | **p** | 0.0157 | 0.0204 | 0.5264 |  |  |  |  |  |
|  |  |  |  |  | **Symbolic Total %** | **Non-Symb COV** |  |  |  |  |  |  |
| M4 | 97 | **R^2^/F:** | 0.12/6.40 | **β** | 0.23 | -0.24 |  |  |  |  |  |  |
|  |  | **p=** | 0.0025 | **p** | 0.0191 | 0.0150 |  |  |  |  |  |  |
|  |  |  |  |  |  |  | **vsWM** |  |  |  |  |  |
| M5 | 97 | **R^2^/F:** | 0.38/18.79 | **β** | 0.11 | -0.11 | 0.54 |  |  |  |  |  |
|  |  | **p=** | <0.0001 | **p** | 0.1889 | 0.2115 | <0.0001 |  |  |  |  |  |
|  |  |  |  |  |  |  | **Reading** |  |  |  |  |  |
| M6 | 97 | **R^2^/F:** | 0.31/14.24 | **β** | 0.08 | -0.20 | 0.47 |  |  |  |  |  |
|  |  | **p=** | <0.0001 | **p** | 0.3915 | 0.0220 | <0.0001 |  |  |  |  |  |
|  |  |  |  |  |  |  | **WISC Vocab** |  |  |  |  |  |
| M7 | 97 | **R^2^/F:** | 0.33/14.93 | **β** | 0.19 | -0.15 | 0.46 |  |  |  |  |  |
|  |  | **p=** | <0.0001 | **p** | 0.0300 | 0.0811 | <0.0001 |  |  |  |  |  |
|  |  |  |  |  |  |  | **Dot Matrix** |  |  |  |  |  |
| M8 | 97 | **R^2^/F:** | 0.36/17.63 | **β** | 0.20 | -0.05 | 0.53 |  |  |  |  |  |
|  |  | **p=** | <0.0001 | **p** | 0.0196 | 0.5643 | <0.0001 |  |  |  |  |  |
|  |  |  |  |  |  |  | **Spatial OR** |  |  |  |  |  |
| M9 | 97 | **R^2^/F:** | 0.27/11.72 | **β** | 0.22 | -0.14 | 0.41 |  |  |  |  |  |
|  |  | **p=** | <0.0001 | **p** | 0.0158 | 0.1225 | <0.0001 |  |  |  |  |  |
|  |  |  |  |  |  |  | **Trail-A RT** |  |  |  |  |  |
| M10 | 97 | **R^2^/F:** | 0.22/8.82 | **β** | 0.22 | -0.21 | -0.32 |  |  |  |  |  |
|  |  | **p=** | <0.0001 | **p** | 0.0187 | 0.0229 | 0.0008 |  |  |  |  |  |
|  |  |  |  |  |  |  | **vsWM** | **Reading** |  |  |  |  |
| M11 | 97 | **R^2^/F:** | 0.48/20.89 | **β** | 0.02 | -0.10 | 0.44 | 0.35 |  |  |  |  |
|  |  | **p=** | <0.0001 | **p** | 0.8081 | 0.1979 | <0.0001 | 0.0001 |  |  |  |  |
|  |  |  |  |  |  |  | **vsWM** | **Reading** | **WISC Vocab** | **Dot Matrix** | **Spatial Or** | **Trail-A** |
| M12 | 97 | **R^2^/F:** | 0.68/23.59 | **β** | 0.06 | 0.04 | 0.18 | 0.25 | 0.23 | 0.27 | 0.26 | -0.16 |
|  |  | **p=** | <0.0001 | **p** | 0.3617 | 0.5467 | 0.0255 | 0.0008 | 0.0016 | 0.0005 | 0.0001 | 0.0122 |
