# Supplementary material for: Cognitive components of a mathematical processing network in 9-year-old children
Source: Dev Sci. 2014 Feb 23;17(4):506–24. doi: 10.1111/desc.12144 (PMC4253132; doi:10.1111/desc.12144)
Supplement: Supplementary file 3 — Methods. Supplementary Methods. [file desc0017-0506-SD3.docx]

**Supplementary Methods**

Maths score

The mathematics tests used were the Mathematics Assessment for Learning and Teaching tests (MaLT) (Williams, 2005)Williams, 2005). The MaLT tests are group-administered written tests. The MaLT tests were developed in accordance with the National Curriculum and National Numeracy Strategy for England and Wales. Test items cover: counting and understanding number, knowing and using number facts, calculating, understanding shape, and measuring and handling data. This test allows for invigilators to read the questions to the children if required to ensure test performance reflects mathematics ability rather than reading proficiency. The MaLT tests were standardized in 2005 with children from 120 schools throughout England and Wales (MaLT 8, α= 0.91; MaLT 9, α=0.93). Tests allowed 45 minutes for completion.

Mathematics was also measured using the Numerical Operations subtest of the Wechsler Individual Achievement Test (WIAT-II UK, Wechsler, 2005). The numerical operations subtest assesses counting, one to one correspondence, numerical identification and writing, calculation (addition, subtraction, multiplication, division) fractions, decimals and algebra.

Mathematics score was a composite of the children's score on the MaLT mathematics test and Numerical Operations subtest.

Reading test.

We used the Hodder Group Reading Test II (HGRT-II) (Vincent & Crumpler, 2007). The HGRT II level 1 was used for Year 3 pupils, and the HGRT II level 2 was used for Year 4 pupils. These multi-choice tests assess children’s reading of words, sentences and passages. The tests were standardized in 2005 with children from 111 schools throughout England and Wales (HGRT II level 1, α=0.96; HGRT II level 2, α=0.95). Each test has two parallel forms which were used in the present study to minimise copying. Tests allowed 30 minutes for completion.

There was also another standardized measure of reading ability (WIAT-II Word Reading subtest) and a standardized measure of phonological decoding (WIAT-II Pseudoword Decoding subtest). Phonological decoding can be considered a subset of phonological awareness skills. Phonological decoding involves identifying and decoding information while phonological awareness also involves manipulating this information (e.g. swapping starting and final phonemes of words).

IQ

In order to estimate IQ we administered a two subtest short-form of the Wechsler Intelligence Scale for Children – 3^rd^ Edition (WISC-III, Wechsler, 1991) which included the Block Design (non-verbal) and Vocabulary (verbal) subtests. This combination of subtests has the highest validity and reliability of the two-subtest forms of the WISC-III (r_tt_ = .91 r=.86; Table L-II, Sattler, 1992).

We also measured children’s non-verbal IQ using Raven’s Coloured Progressive Matrices (Raven’s – Educational: CPM, Raven, 2008).

Working memory

We assessed working memory using five subtests from the Automated Working Memory Assessment (AWMA, Alloway, 2007). The AWMA is a computer administered battery of tests which assess verbal short term memory (STM) and visuo-spatial STM and working memory (corresponding to the phonological loop, visuo-spatial sketch pad and central executive components of Baddeley and Hitch’s (1974) model of working memory, respectively).

Verbal working memory

*Short-term memory (STM):* Phonological Loop: The Digit Recall subtest requires children to listen to a sequence of digits and recall them. Because we did not want to confound STM performance with a general difficulty with mathematical information in our weaker maths group we also measured verbal STM using the Word Recall subtest. This subtest requires children to listen to a series of real words and to recall them.  In both verbal STM subtests the item is scored as correct if the child recalls the series in the correct order.

*Central executive:* Listening span. The Listening Span subtest requires children to listen to sentences and to decide whether the sentences are true or false. Children then recall the final word of the each sentence. Where two or more sentences occur in the same trial, the children must process the content of each sentence. At the end of the final sentence the child must recall the final words of each sentence in the same order as they were presented. An item is scored as being correctly processed if the child correctly decides whether the sentence is true or false. An item is scored as being correctly recalled if the child recalls the final words of the sentences in correct order.

Visual working memory

*Short-term memory (STM;* Visuo-spatial sketch pad): The Dot Matrix subtest requires children to view the position of a red dot in a series of four by four matrices and to repeat the sequence by tapping on the computer screen. An item is scored as correct if the child recalls the sequence in the correct order.

*Central executive:* Odd-one-out (OOO). In the OOO task three abstract shapes are presented on the computer screen. One of the shapes differs from the other two shapes and children must identify the odd shape by tapping it on computer screen. The shapes then disappear and the child must recall where the odd shape was located by tapping one of three empty boxes on the computer screen. Where two or more sets of shapes occur in the same trial, the children must identify the odd shape in the first set, then in the subsequent set(s). At the end of the final set of shapes, the shapes disappear and the children must recall the locations of the odd shapes in the same order as presented. An item is scored as being correctly processed if the child correctly identifies the odd shape. A set is scored as being correctly recalled if the child recalls the locations of the odd shapes in the correct order. A set is also scored as being correctly recalled if the child misidentifies the odd shape but correctly recalls the location of the misidentified shape during recall.

Standardized *recall* scores were measured for all subtests ('Digit Recall', 'Word Recall', 'Dot Matrix', 'List Recall Storage' and 'OOO Recall' in Results) and standardized *processing* scores were measured for OOO and Listening Recall ('OOO Processing' and 'List Recall Processing' in Results). Raw scores were also measured for OOO processing/ recall and Dot Matrix.

Socio-economic status (SES)

SES was estimated from parental occupations and education levels which were obtained via a questionnaire included with the consent form and information pack. The questionnaire asked parents for the highest level of education completed. Education levels were classified on a 7 point scale: 1. Doctorate Degree, 2. Master's Degree, 3. Bachelor's Degree, 4. Some University, 5. A Level or equivalent, 6. GCSE or equivalent and 7. Less than secondary school. Professional diplomas were also coded as 4. and postgraduate qualifications other than Master's degrees were coded as 3.

Parents' occupations were scored according to the Standard Occupational Classification 2010 (retrieved from: http://www.ons.gov.uk/ons/guide-method/classifications/current-standard-classifications/soc2010/index.html). Occupations were coded according to this classification scheme: 1. Managers, Directors and Senior Officials, 2. Professional Occupations, 3. Associate Professional and Technical Occupations, 4. Administrative and Secretarial Occupations, 5. Skilled Trades Occupations, 6. Caring, Leisure and Other Service Occupations, 7. Sales and Customer Service Occupations, 8. Process, Plant and Machine Operatives, 9. Elementary Occupations. We included an additional category (0) to classify parents that were unemployed.

Trail Making Task

The Trail Making Task (TMT) A and B, measures of executive functioning and task-switching, were administered. In the TMT A the children were required to connect a series of numbered circles which were positioned across an A4 page. Children were instructed to connect the circles in sequence without removing their pencil from the paper, if possible, and to connect the circles as quickly as they could. The time to complete the sequence was recorded and the test was scored with 2 points if it was completed correctly or if self-corrected errors were made. A score of 1 was given for sequences that were mostly correct but for one uncorrected error and a score of 0 was given if the sequence was clearly incorrect or if the path could not be determined.

In the TMT B the children were presented with a similar page of numbered and lettered circles, and were required to connect the circles in sequence, however they were required to switch between numbers and letters (e.g., 1 - A - 2 - B - 3 - C and so on). The time to complete the sequence was recorded and the accuracy of the responses were scored using the same scoring scheme as for the TMT A.

Finger knowledge

Children were presented with an A4 chart depicting an illustration of a left and right hand with finger labels in order to familiarise the children with the names of the different fingers. We used the labels “thumb”, “pointer”, “middle”, “ring” and “pinky”. The children were told that they needed to know the names of the fingers because they were going to be asked to close their eyes and they would have to say which finger the experimenter had just touched. Once children were confident that they knew the names of the fingers they were asked to close their eyes and the experimenter then touched the back of each of their fingers, in a randomised order, using the eraser on the end of a pencil. Children's responses were recorded using a digital voice recorder for later scoring. If the child appeared to have difficulty recalling the names of the fingers (e.g., saying “I've forgotten the name”) the experimenter allowed the child to open their eyes to refer to the finger label chart in order to answer, but the experimenter gave no indication of which finger had been touched and did not continue with the test until the children had closed their eyes again. Responses were scored with 1 point for each correct response.

Line bisection

In the line bisection task, a measure of spatial bias, children were required to bisect 16 lines. Children were instructed to mark where they thought the "halfway" point of each line was.

Lines were 1pt weight and ranged in length between 114 and 233 mm. Lines were presented individually on strips of paper in order to remove any spatial bias caused by the simultaneous presentation of lines on the same page. Four lines were centrally aligned with respect to the midline of the strips of paper, six lines were aligned to the left and six lines were aligned to the right. The lines that were aligned to the left or right of the midline were matched so that they were shifted an equal distance (the shift ranged between 10 and 50 mm from the midpoint).

The total time to bisect the 16 lines was recorded. The distance from the leftmost point of the line to the point where the children made their midpoint estimate was measured to the nearest mm using a ruler. The difference between the actual midpoint and the estimate was calculated and recorded for each item.

Mental rotation

Our mental rotation task was based on Quaiser-Pohl's Picture Rotation Test (Quaiser-Pohl, 2003). Three separate worksheets with different stimuli types (objects/animals, letters and hands) were presented to the children in a counterbalanced order; each worksheet had seven items.

Stimuli

The object/animal stimuli were obtained from:

<http://www.nefy.ucl.ac.be/facecatlab/stimuli.htm>

These stimuli are based on the widely used line drawings of Snodgrass and Vanderwart (1980). Object and animal pictures familiar to children, with no rotational or reflective symmetry were selected.

For the letter rotation task seven letters with no reflective or rotational symmetry were used (F, G, J, K, L, P, R,). Letters were presented in uppercase and in Arial font.

The stimulus set for the hand stimuli was kindly provided by Professor Lawrence Parsons (Parsons, 1994), which comprised of line drawings of left and right hands “viewed from five cardinal perspectives” (p. 711). The views of the different hands were front, back, and two other more unusual views e.g. side view for each hand (see Parsons, 1994, for stimuli).

For each item within a worksheet, a target stimulus was presented, along with three comparison stimuli, two of which are mirror images (distractors) and one was identical to the target. All three comparison images are rotated by various angles, and the child has to identify and circle the stimulus identical to the target. Rotation of the comparison stimuli was between 60 and 300 degrees (at intervals of 60 degrees resulting in five levels: 60, 120, 180, 240, 300). The position of the identical stimulus within the three comparison pictures was chosen at random, as was the angle of rotation at all three positions (under the condition that the same angle did not occur twice).

Procedure

The child was instructed to circle “the picture that is facing the same way when you imagine them all the right way up” and to complete all seven items on each page as quickly as possible. The children's total response time to complete all seven items was timed separately for the three pages.

Spatial Symmetry

In the symmetry drawing task, a measure of knowledge of spatial symmetry, children were presented with two pages which contained six half drawn shapes against a grid background with a line of symmetry indicated with a dashed line. Children were required to draw the other half of the shape for each item. The shapes increased in difficulty from a simple rectangle to a series of steps. Shapes (and lines of symmetry) were presented vertically on one page and horizontally on the other. Children were instructed to complete the other half of the shape and if they found an item too difficult to move onto the next shape or the next page. Half of the children received the horizontally-oriented shapes first and the other half of the children received the vertically-oriented shapes first. The total time to complete the 12 shapes was recorded and the accuracy of items was scored with one point for every correct line segment.

Spatial Orientation Task

We used six items from the Object Perspective Test (Kozhevnikov & Hegarty, 2001) as a measure of children's spatial orientation ability. In this task children were presented with a map containing different items such as a tree, car, cat and traffic light. Children were required to imagine themselves in this space, and to imagine they were standing next to one item, and facing another item. The children were then required to estimate the direction of a third item (e.g., "Imagine you are standing next to the cat and facing the house. Point to the traffic light"). Children were given a page containing a circle which was blank except for a line oriented north indicating the location of the item the child was to imagine facing. The centre point of the circle indicated the object that the children were to imagine standing next to. Children were required to indicate the direction of the third object by drawing a line from the centre of the circle to its edge. Children completed a practice question before six experimental questions. Responses were measured using a protractor and responses were recorded as correct if they fell within ±20 degrees of the correct location. We used this range because the location at which the child imagined they were next to the object was subjective, for example, children may have imagined themselves standing to the left or right of the object or at the centre of the object, which consequently affects the angle at which they would have located the third object. Correct responses received a score of 1, with a maximum of 6 points available.

Computerized Experimental Tasks

The following tasks were presented by the Presentation program of Neuro-behavioral Systems using a laptop computer. Unless described otherwise, reaction time and accuracy were recorded for all trials.

Simple RT

In the task designed to measure baseline simple RT, children were instructed to press the space bar with their dominant hand as quickly as they could when a white box appeared on the screen. The white box, measuring 100 x 100 pixels, appeared at the centre of the screen for 3000 ms or until the child made a response. The delay before the square appeared was 1,000, 2,500 or 4,000 ms. The children received 6 practice trials before completing two blocks of 30 trials.

Sustained Attention

Children were required to attend to a stimuli stream and to detect a target sequence. Seven letters of the alphabet (from 'A' to 'H') were used. White letters on black background followed each other in rapid succession (300 ms). One letter was presented for 300 ms. The task was to continuously attend the stimuli stream and detect and indicate with a button press if the series of 'A B C' was seen, but not to respond if other combinations including A, B and C were presented, e.g. 'A C B' or 'A B D' etc.

There were three types of letter series:

1. targets (A B C)
2. 'deceivers'
   1. series beginning with A, then B followed by a letter out of the target range [D to H]
   2. series beginning with an A and ending with a C, but with another letter in the middle [D to H])
   3. series beginning with a letter D to H but ending with B and C
3. non-targets: random combinations of one letter from the target range [A B C] and two letters out of the target range [D to H]

The series were pseudo-randomized so that more than two target series could not follow each other, however, there was no break in between the letter 'triads'; letters were presented in one continuous stream.

There were 80 of each type of letter series (3 letter types x 80 triads x 3 letters in total).

Children completed a practice block of 24 triads, in which the letters in the target series were highlighted in red and underscored with yellow. These indicators served to familiarise the children with the target sequence during practice and were removed for the experimental block.

The number of hits and misses for targets, and the RT for target hits was recorded. Furthermore the number of correct rejections and false alarms for deceivers and non-target trials was recorded.

Stop Signal Task

In the Stop Signal Task, a measure of response inhibition, a white arrow, pointing left or right, was shown for 500 ms on a black background in the middle of the screen. The arrow was either followed by a sound, the stop signal, or there was no sound. The time delay until the stop sound was dynamically varied between 0 and 1000 ms (see below). The ratio of 'go' and 'stop' trials was 2:1. Trials were separated by the presentation of an eye for 500 ms.

On 'go' trials, participants had to press the left 'Ctrl' button on the keyboard if the arrow pointed left and the right 'Ctrl' button if the arrow pointed to the right. On 'stop' trials the stop signal was played after the arrow, indicating that participants should withhold their response to the arrow. The stop signal sound was a 500Hz tone with a 10 ms rise- and fall time which played for 500 ms.

The time delay until the stop signal (sound) after the presentation of the arrow was set from trial to trial, depending on how the participants performed on the previous 'stop' trial. After an unsuccessful 'stop' trial (i.e. when they could not stop after the stop signal) the delay was decreased by 50 ms; after a successful 'stop' trial the delay was increased by 50 ms. Note that a shorter stop signal delay after the arrow makes the task easier because participants may not have begun to initiate their motor response yet so it is easier to inhibit, whereas a longer delay makes the task more difficult because the longer the delay the more likely the participant will have initiated their response.

Participants completed 12 practice trials before completing three experimental blocks of 60 trials. Stimuli series were randomized for each participant separately. For each trial we measured RT, sstRT (defined as the RT - average stop signal delay), and the number of times the child responded to the arrow incorrectly.

The following three described tasks were measures of the number sense.

Subitizing

An array of dots appeared on the screen and the children were instructed to say the number of dots as quickly as possible. The number of dots in each array ranged between one and six. For each set size, an array was constructed using dots which were one of five sizes; the diameter of each dot within an array was 5, 7.5, 10, 12.5 or 15 mm. The dot stimuli were black, were presented in canonical and, where possible, non-canonical arrangements, in the centre of the screen against a white background.

Children's reaction times were measured using a voice key. After the children responded the experimenter also entered the child's answer using an external numerical keyboard, which was necessary for scoring the accuracy of responses, and advanced the experiment to the next trial. Children completed six practice trials (one of each set size) before completing two blocks of 30 trials (each block contained all combinations of the six set sizes and five dot sizes). Subitizing range was defined as 1-3 dots and counting range was defined as 4-6 dots. This division was confirmed by examining reaction times which showed the characteristic switch between subitizing and counting range processes between set sizes of 3 and 4. Reaction times (and standard errors) in milliseconds for 3 to 6 items, respectively: 772±2; 834±2; 942±2; 1350±4; 1736±5; 2278±7 ms. Consecutive differences between RTs for the 3 to 6 items in milliseconds (from 1/2 to 5/6): 62; 108; 408; 386; 542 ms.

Symbolic Magnitude Comparison task (Distance Effect)

This task was similar to the magnitude comparison task used by Soltész and Szűcs (2009). In the symbolic magnitude comparison task participants decided whether visually presented digits (1, 4, 6 or 9) were smaller or larger than 5. White digits with a font-size of 40 were presented in the middle the computer screen on a black background. Trials started with a picture of an eye shown for 200 ms. After 1000 ms a digit was shown for 3000 ms or until the child made a response. 400 ms passed before the next eye appeared. Children pressed a button on the keyboard with their left hand if the number was smaller than 5 and another button with their right hand if the number was larger than 5. Two blocks of 40 stimuli were presented, preceded by 8 practice stimuli before the first block.

Nonsymbolic magnitude comparison task (non-symbolic ratio effect)

This task was used by Soltész et al. (2010). Black dots on white background were used as stimuli. Two sets of dots were presented simultaneously on the computer screen. The sets were separated by 7.5 cm, and were visually easily distinguishable from each other. The overall envelope (corresponding to contour length in Rouselle et al., (2004)) of a set was kept constant at 9×9 cm, as overall envelope has been found to help children even in conditions where overall surface is incongruent with number (Barth et al., 2005). Children’s task was to find out which set contains more dots and press the button on the side of the larger set. Response side was counterbalanced.

The size of dots was constant within set and varied between sets. The individual size of dots and the pattern of dots were randomly varied through pairs of sets. Sets with the same number of items never had the same dot size. Only numerosities above the subitization range were used in order to exclude that object-based attention (or the object file system, Simon, 1997; Kahneman, Treisman & Gibbs, 1992; Uller et al., 1999; Huttenlocher et al., 1994) would be used to complete the comparison task.

The following factors were taken into consideration: (1) The ratio of the number of dots in the two sets. (2) The numerical distance between the number of dot in the two sets. (3) The type of the physical control variable. (4) The congruity of physical control variables and numerosity. (5) The overall numerical sum of items in a display. The ratios and numerical distances for all combinations of numerosities are summarized in Supplementary Methods Table 1. Accordingly, all number pairs used are shown in Table 1. There was no extreme large numerical distance for the 2:3 ratio because the sum of items would have been much larger than in other conditions (at least 16:24, sum is 40; or 20:30, sum is 50). Rather, we decided to keep the sum of items for the largest numerical distances in the 1:2 and 3:5 ratio conditions to be approximately equal to the sum of items in the distance 6 condition in the 2:3 ratio condition. This allowed for checking for ratio effects independent from the overall sum of items in the display.

Two different physical variables were manipulated as controls: the overall surface (hence, luminance) and the overall circumference (sum of the individual items’ circumferences) of the dot groups. These two physical controls were intermixed during stimulus presentation. The ratios of the overall physical sizes (surface in half of the trials and circumference in other half of the trials) of dot sets were congruent or incongruent with the numerical ratio of the dot sets. In the congruent condition the more numerous set was larger in overall physical size than the less numerous set. In the incongruent condition the more numerous set was smaller in overall physical size than the less numerous set. Congruent and incongruent trials were pseudo-randomly intermixed (no more than three of each could follow in a sequence).

In each trial the ratio of perceptual features of the two dot patterns was kept the same as their numerical ratio. This was done because if the ratio of the surfaces or circumferences within a set pair were not in accord with the ratio between the numbers in the set pair, the influence of perceptual variables would differ among numerical ratios. For example, if the ratio between the numbers were 1:2 and the ratio between perceptual variables were 2:3, the perceptual difference would be less salient than the numerical difference. This would result in better numerical discrimination performance solely because physical variables would be less distractive. Similarly, if the perceptual ratio were 1:2 and the numerical ratio were 2:3, the perceptual difference would be more salient. Our design avoided this problem.

In order to investigate the effects of numerical distance and ratio independent from each other, separate analyses were performed on number pairs varying in numerical distance while ratio was kept constant, and on number pairs with identical numerical distances and with varying ratio.

Each trial consisted of a fixation sign (brackets) shown for 500 ms followed by a 1000 ms blank period, and the stimulus stayed on the screen until the subject gave a response. The offset of the stimulus was followed by a 1000 ms blank period. There were 4 blocks of 32 stimuli. The experiment was preceded by 12 practice stimuli.

**Supplementary Results**

Variance Inflation Factor in main models

The range of VIF values computed from the correlation table of variables listed in **Supplementary Table 3**. was 1.19-1.81; and for the variables listed in **Table 4**: 1.34-1.86. This confirms that muti-collinearity problems did not affect our analyses.

Checking regression assumptions

Our best model **(Table 4; M3** and **Fig. 2C.)** conformed well to the assumptions of multiple linear regression. **Supplementary Figure 1A.** shows regression residuals in function of predicted scores and Lowess means and standard deviations with a running window of 20 data points. First, residuals were centred on the zero line, well conforming to the linearity assumption of linear regression. Second, the variance of residuals around the regression line was consistent (homoscedasticity). The only slight deviation from assumptions was that the distribution of residuals was not perfectly normal; Kolgomorov-Smirnov statistics = 0.155; p=0.0163; (**Supplementary Figure 1B-C**). However, the deviation was very mild (**Supplementary Figure 1B-C.**); the normality assumption is less important with large sample sizes (Cohen et al. 2003) and the bootstrap procedure provides sufficiently robust estimates of parameters and significance levels when taking into account variability in the data. Further tests confirmed that outliers did not influence regression outcomes (**Supplementary Figure 1D-F.**). Most leverage values were well within the acceptable range. (Critical leverage value: 2×(number of model parameters including constant)/Number of observations)=2×7/98=0.1428). There was one case with relatively high leverage. However, when examining Cook's distance which gives an indication of the actual influence of each particular case on regression outcomes it became evident that there were no cases with particularly high individual influence (critical value = 1; maximum value in our data < 0.1) and the case with the highest leverage had very small influence (**Supplementary Figure 1F.**).

Predicting number sense variables

While it was not the main objective of the current study it is theoretically important to identify potential predictors of number sense variables. As shown in **Supplementary** **Table 2.** Symbolic comparison total accuracy was moderately related to WISC Vocabulary, Dot Matrix, verbal WM, Spatial Orientation and especially, sustained attention. First, these variables were entered into a regression as predictors of symbolic comparison accuracy. Second, Spatial Orientation, WISC Vocabulary and Sustained attention were used as predictors. Third, only Spatial Orientation and Sustained Attention were used. In all these analyses Sustained Attention emerged as the only significant predictor (in all three analyses: N=97; 0.457≤β≤0.49; p<0.0001. 0.19≤R^2^≤0.21; 4.7≤F≤10.8). Fourth, the model for reading performance was used with predictors WISC Vocabulary, Phonological Decoding, Sustained attention. This model gave the best fit (overall model R^2^=0.24; F=9.19; p<0.0001. Phonological Decoding: β=0.231; p=0.029. Sustained attention: β=0.41; p<0.0001. WISC Vocabulary: n.s.). Fifth, when using the best model predicting math the Phonological Decoding score was the only significant predictor (β=0.321; p=0.005. Overall model R^2^=0.14; F=2.44; p=0.0315).

In another set of analyses symbolic comparison COV was predicted by the above 5 models. Sustained attention was the only significant predictor (in all four models including sustained attention: -0.32≤β≤-0.3; p<0.008. Overall model: 0.12≤R^2^≤0.14; 2.94≤F≤6.45; 0.0028≤p≤0.0078).

An additional analysis used Reading, visual WM, Stop Signal and Sustained attention as predictors of symbolic comparison RT because these variables were correlated with symbolic comparison RT. Again, only Sustained Attention emerged as a predictor (β=-0.451; p<0.0001. Overall model R^2^=0.22; F=5.98; p=0.0003).

In further analyses subitizing COV was predicted by the above 5 models. Sustained attention was the only significant predictor (In all four models including sustained attention: -0.282≤β≤-0.265; p<0.026. Overall model: 0.09≤R^2^≤0.10; 2.07≤F≤2.90; 0.0111≤p≤0.0393). None of the above models could significantly predict total subitizing performance.

None of the above models could significantly predict total performance on the non-symbolic comparison task. When trying to predict non-symbolic comparison COV only the Dot Matrix task emerged as a significant predictor in each of the two of the above five models which included it (-0.345≤β≤-0.311; p≤0.008). Spatial Orientation emerged as a marginal predictor in each of the two of the above five models which included it (-0.198≤β≤-0.185; p≤0.083). When including only Dot Matrix (β=-0.323; p=0.001) and Spatial Orientation (β=-0.171; p=0.081) as predictors the β values were similar to the above and the overall model fit was moderate: R^2^=0.16; F=8.87; p=0.0003.

**Supplementary Figure captions**

**Supplementary Figure 1.**

Checking regression assumptions (for the model in **Table 4; M3** and **Fig. 2C.)**. (A) Regression residuals in function of predicted scores and Lowess means and standard deviations with a running average window of 20 data points. (B) Plotting residuals against the normal distribution. (C) Quantile-Quantile plot of residuals. (D) Leverage of residuals. (E) Cook distance of residuals. (F) Cook distance vs. leverage.

**Supplementary References**

Alloway, T.P. (2007). *Automated Working Memory Assessment (AWMA).* London: UK: Pearson Assessment.

Baddeley, A.D., Hitch, G.J. (1974). Working memory. In: Bower GA, Editor. *The Psychology of Learning and Motivation*. New York: Academic Press, p. 47–89

Barth, H., La Mont, K., Lipton, J., & Spelke, E.S. (2005). Abstract number and arithmetic in preschool children. *PNAS U S A, 102(39),* 14116-14121.

Huttenlocher, J., Jordan, N., & Levine, S.C. (1994). A mental model for early arithmetic. *Journal of Experimental Psychology: General, 123*, 284-296.

Kahneman, D., Treisman, A., & Gibbs, B.J. (1992). The reviewing of object files: object-specific integration of information. *Cognitive Psychology, 24*, 175-219.

Parsons, L. (1994). Temporal and kinematic properties of motor behavior reflected in mentally simulated action. *Journal of Experimenal Psychology: Human Perception and Performance, 20,* 709-730.

Quaiser-Pohl, C. (2003). The mental cutting test “Schnitte” and the picture rotation test-two new measures to assess spatial ability. *International Journal of Testing, 3,* 219-231.

Raven J. (2008). *Raven’s - Educational: Coloured Progressive Matrices (CPM).* London: Pearson Assessment.

Rousselle, L., Palmers, E., & Noël, M-P. (2004). Magnitude comparison in preschoolers: What counts? Influence of perceptual variables. *Journal of Experimental Child Psychology, 87,* 57-84.

Sattler J. (1992). *Assessment of Children, 3rd Edition.* San Diego: Jerome Sattler.

Simon, T.J. (1997). Reconceptualizing the origins of number knowledge: A non-numerical account. Cognitive Development, 12, 349-372.

Snodgrass, J., & Vanderwart, M. (1980). A standardized set of 260 pictures: Norms for name agreement, image agreement, familiarity, and visual complexity*. Journal of Experimental Psychology: Human Learning, 6,* 174–215. doi:10.1037/0278-7393.6.2.174

Soltész, F., & Szűcs, D. (2009). An electro-physiological temporal principal component analysis of processing stages of number comparison in developmental dyscalculia. *Cognitive Development, 24(4)*, 473–485. doi:10.1016/j.cogdev.2009.09.002

Soltész, F., Szűcs, D., & Szűcs, L. (2010). Relationships between magnitude representation, counting and memory in 4- to 7-year-old children: a developmental study. Behavioral and Brain Functions, 6, doi:10.1186/1744-9081-6-13.

Soltész, F., Goswami, U., White, S., & Szűcs, D. (2011). Executive function effects and numerical development in children: Behavioural and ERP evidence from a numerical Stroop paradigm. *Learning and Individual Differences, 21(6),* 662–671. doi:10.1016/j.lindif.2010.10.004

Szűcs, D., Soltész, F., & White, S. (2009a). Motor conflict in Stroop tasks: direct evidence from single-trial electro-myography and electro-encephalography. *Neuroimage, 47(4),* 1960–73. doi:10.1016/j.neuroimage.2009.05.048.

Szűcs, D., Soltész, F., Bryce, D., Whitebread, D. (2009b). Real-time tracking of motor response activation and response competition in a Stroop task in young children: A lateralized readiness potential study. *Journal of Cognitive Neuroscience, 21(11),* 2195–206. doi:10.1162/jocn.2009.21220

Uller, C.M., Carey, S., Huntley-Fenner, G., & Klatt, L. (1999). What representations might underlie infant numerical knowledge. *Cognitive Development, 14*, 1-36.

Vincent, D., & Crumpler, M. (2007). *Hodder Group Reading Tests 1-3 (II.).* London: UK: Hodder; Education.

Wechsler, D. (1991). *Wechsler Intelligence Scale for Children -Third Edition (WISC-III).* San Antonio, TX: Psychological Corporation.

Wechsler, D. (2005). *Wechsler Individual Achievement Test - Second UK Edition (WIAT-II UK).* London: UK: Harcourt Assessment.

Williams, J. (2005). *Mathematics Assessment for Learning and Teaching.* London: UK: Hodder Education.
